# Patatin-related phospholipase pPLAIIIδ influences auxin-responsive cell morphology and organ size in *Arabidopsis* and *Brassica napus*

**DOI:** 10.1186/s12870-014-0332-1

**Published:** 2014-11-27

**Authors:** Yanni Dong, Maoyin Li, Peng Zhang, Xuemin Wang, Chuchuan Fan, Yongming Zhou

**Affiliations:** National Key Laboratory of Crop Genetic Improvement, Huazhong Agricultural University, Wuhan, China; Donald Danforth Plant Science Center, St Louis, Missouri USA

**Keywords:** Auxin, pPLAIIIδ, Cell morphology, Phospholipase, Ethylene, Phosphatidic acid

## Abstract

**Background:**

The members of the patatin-related phospholipase subfamily III (pPLAIIIs) have been implicated in the auxin response. However, it is not clear whether and how these genes affect plant and cell morphogenesis. Here, we studied the roles of the patatin-related phospholipase pPLAIIIδ in auxin-responsive cell morphology and organ size in *Arabidopsis* and *Brassica napus*.

**Results:**

We show that overexpression of *pPLAIIIδ* inhibited longitudinal growth but promoted transverse growth in most organs of *Arabidopsis* and *Brassica napus*. Compared to wild-type plants, *pPLAIIIδ*-KO plants exhibited enhanced cell elongation in hypocotyls, and *pPLAIIIδ*-OE plants displayed broadened radial cell growth of hypocotyl and reduced leaf pavement cell polarity. For the hypocotyl phenotype in *pPLAIIIδ* mutants, which resembles the “triple response” to ethylene, we examined the expression of the *ACS* and *ACO* genes involved in ethylene biosynthesis and found that *ACS4* and *ACS5* were up-regulated by 2.5-fold on average in two OE lines compared with WT plants. The endogenous auxin distribution was disturbed in plants with altered *pPLAIIIδ* expression. *pPLAIIIδ*-OE and KO plants exhibited different sensitivities to indole-3-acetic acid-promoted hypocotyl elongation in both light and dark conditions. Gene expression analysis of auxin-induced genes in the dark showed that OE plants maintained a higher auxin response compared with WT and KO plants after treatment with 1 μM IAA for 12 h. Following treatment with 10 μM IAA for 30 min in the light, early auxin-induced genes were significantly up-regulated in two OE plant lines.

**Conclusions:**

These data suggest that the *PLAIIIδ* gene plays an important role in cell morphology and organ size through its involvement in the regulation of auxin distribution in plants.

**Electronic supplementary material:**

The online version of this article (doi:10.1186/s12870-014-0332-1) contains supplementary material, which is available to authorized users.

## Background

The patatin-related phospholipase A proteins consist of three subfamilies, pPLAI, pPLAII (α, β, γ, δ, ε), and pPLAIII (α, β, γ, δ), based on their sequence similarity [1]. This group of enzymes hydrolyses phospholipids and galactolipids [2]. The plant-specific *pPLAIII* subfamily differs from the other patatin-related phospholipases in several aspects, including the intron/exon structures associated with intron loss during evolution, an altered esterase box (GXGXG), and the lack of the Leu-rich repeat (LRR) motif present in pPLAI [1].

Plant pPLAIII proteins participate in signal transduction, membrane remodelling, and lipid metabolism through the production of various fatty acids and lysophospholipids. Lysophosphatidylethanolamine (LPE) delayed fruit maturation and leaf senescence in tomato due to the enhanced stability of the cell membrane [3]. Free fatty acids and/or lysophospholipids may function as the second messengers in auxin signal transduction in zucchini [4]. Treatments of *Arabidopsis* seedlings with free fatty acids 18:2 and 18:3 or LPE and lysophosphatidylcholine (LPC) inhibited auxin-regulated primary root growth [5]. The production of LPC in intact cells could quickly result in the activation of H^+^-pumps, which contribute to the auxin-induced corn coleoptile elongation [6]. Lysophosphatidic acid (LPA) stimulated the activation of phospholipase D (PLD) to generate PA, which has been identified as a vital signalling molecule during pathogen infection, drought, salinity, wounding, and cold stress [7].

pPLAIII proteins may play important roles in the hormone-mediated development of plant organs. All four genes in the *pPLAIII* subfamily (α, β, γ, δ) have been proven to be activated by auxin [8]. A gain-of-function mutant (*STURDY*) of *pPLAIIIδ* exhibited a stiffer floral stem, thicker leaves, and larger seeds [5,9]. Hormone-related phenotypes in root growth, hypocotyl photomorphogenesis, lateral root initiation, and root hair development have also been observed in *pPLAIIIδ*- and *pPLAIIIβ-*knockout mutants [5,8]. Research on *pPLAIIIβ* has suggested that these aberrant organs may be a result of modified cell shape in the mutants [5].

In plants, the control of cell shape depends on polarised cell expansion, which relies on the establishment and maintenance of an intracellular polarity signal through cytoskeletal dynamics and vesicle trafficking [10]. As a master regulator, auxin exhibits pleiotropic effects on flexible cell morphogenesis, both directly and indirectly [11,12]. The function of the auxin polar transport system relies on the directional cellular localisation of the auxin efflux carrier PIN-FORMED (PIN) proteins [13], the auxin influx carrier AUX1/LIKE-AUXIN (AUX1/LAX) proteins [14], and the ATP-dependent multi-drug resistance/P-glycoprotein (MDR/PGP)-type ABC transporters [15]. The vesicle trafficking, phosphorylation, and dephosphorylation of PINs result in their diverse subcellular distributions in various cell types [16], such as the basal localisation of PIN1 in both shoots and roots, the apical localisation of PIN2 in root epidermis cells, and the lateral polarity of PIN3 in shoot endodermis cells [17-19]. The different subcellular localization of PINs guided the auxin flow to cause polydirectional cell growth [20]. Integration of various hormone signals occurs during cell morphogenesis in various cell types. Ethylene is considered to constitute the cross-talk junction of the strigolactone and auxin pathways in mediating root hair elongation [21]. Under ACC treatment, a *PIN3* loss-of-function mutant was shown to display a strongly reduced response to ACC in hypocotyl elongation [22]. Auxin and cytokinin signalling through ROP GTPase-dependent pathways have opposite effects on coordinating the formation of the interdigitated pattern of leaf pavement cells [23]. However, the mechanism that regulates the formation of these phenotypes in *pPLAIII* mutants remains to be determined.

Here, we studied the roles of pPLAIIIδ in plant development through the characterisation of the *pPLAIIIδ* loss-of-function and gain-of-function mutants. Altered expression of *pPLAIIIδ* affects plant growth and size through modifications of cell expansion and elongation. Such phenotypic changes are concurrent with modified lipid profiles. Our data therefore show that the *pPLAIIIδ* gene plays an important role in the growth and development of plant organs, cell morphogenesis, and auxin signal transduction in *Arabidopsis* and its close relative *Brassica napus*.

## Results

### Temporal and spatial expression patterns of *pPLAIIIδ*

**Figure 1:**
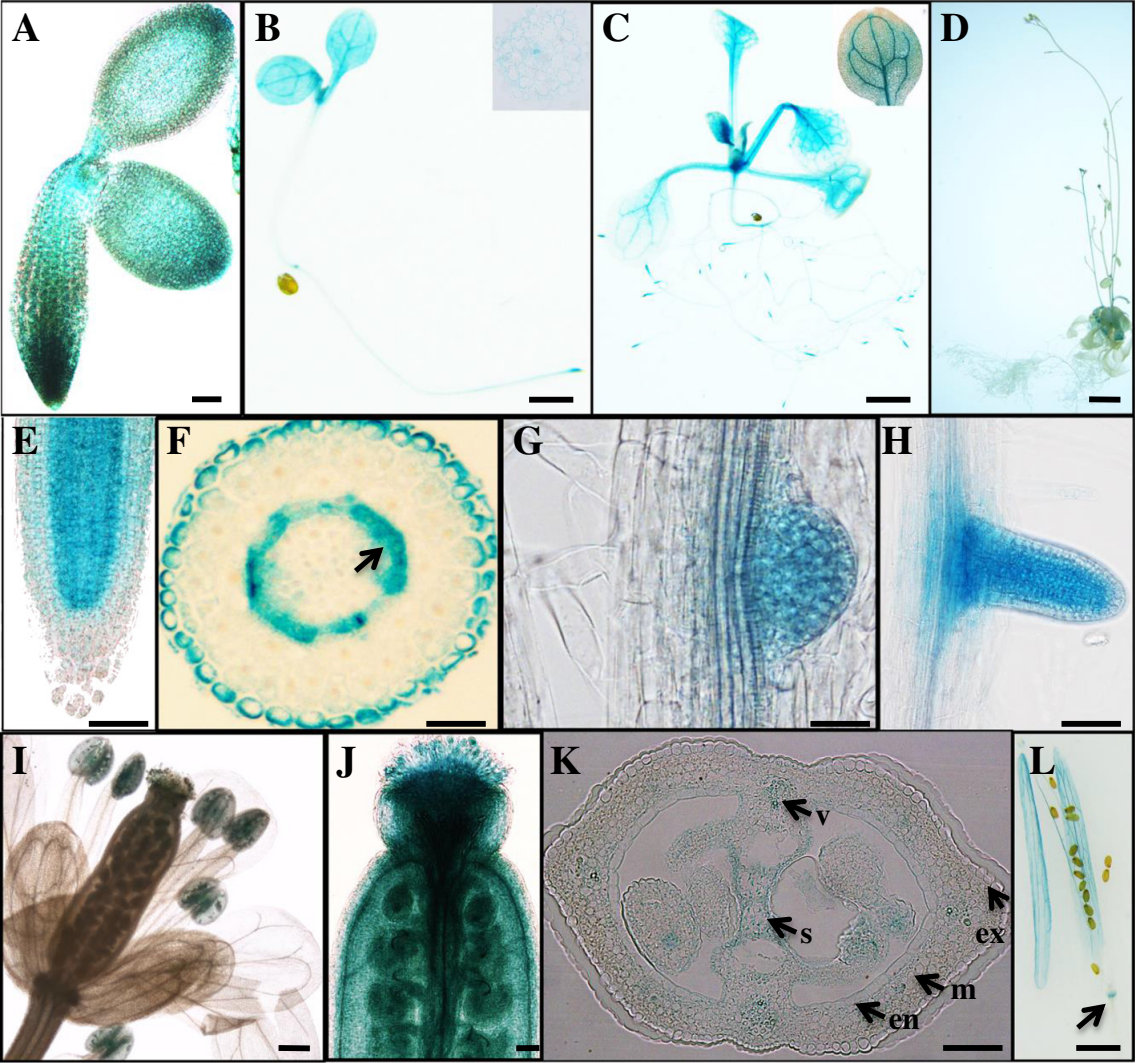
**GUS activity in transgenic** ***Arabidopsis*** **plants of** ***pPLAIIIδ::GUS*** **fusions. (A)** A seed sprouting after 24 h. **(B)** 7-day-old seedling (a magnified image of a cross-section of the hypocotyl). **(C)** 15-day-old seedling (a magnified image of a leaf). **(D)** 5-week-old plant. **(E)** pPLAIIIδ expressed in the elongation and meristem zones of the primary root. **(F)** Cross-section of the primary root. **(G)** Lateral root at stage VI of lateral root development. **(H)** Lateral roots emerging from the primary root. **(I)** Flowers. **(J)** The gynoecium 48 h after hand-pollination. **(K)** Cross-section of an immature silique. **(L)** The mature silique. v, vascular bundles; s, septum; ex, exocarp; m, mesocarp; en, endocarp. Bars =10 μm **(E to H, J)**, 100 μm **(A, I, K)** or 1 cm **(B, C, D and L)**.

Our previous study showed that the *pPLAIIIδ* gene is expressed in various tissues [5]. To gain further insight into how *pPLAIIIδ* expression may affect the growth and development of plant organs, independent *Arabidopsis* transformants of *pPLAIIIδ*::*GUS* plants were generated and examined throughout plant development. At early stages, GUS staining was observed in the seedlings, especially in roots (Figure 1A-C, E), hypocotyls (magnified image in Figure 1B), vascular tissues of leaves, and the stem apical meristem (Figure 1A-C). The GUS staining became weaker at the flowering stage (Figure 1D). Cross-sections of the primary root tip revealed that *pPLAIIIδ* was specifically expressed in the epidermis and endodermis and pericycle cells (Figure 1F), and the developing lateral roots showed intense GUS staining (Figure 1G and H). These profiles were consistent with the microarray data from Genevestigator (see Additional file 1). The open flowers (Figure 1I) and ovules, valves, septum, and stigma after pollination for 48 h were positively stained (Figure 1J). During the development of the silique, *pPLAIIIδ* was mainly expressed in vascular bundles, as well as the septum, endocarp, mesocarp, and exocarp (Figure 1K), and there was no visible staining in mature siliques except for the coat and the junction point of the silique and pedicel (Figure 1L, arrow). Quantitative PCR showed that *pPLAIIIδ* expression was significantly higher in the roots than in the leaf, stem, flower, silique, and seed (see Additional file 1). These findings showed that *pPLAIIIδ* was expressed in various tissues during the development and growth of plant organs, with preferential expression of this gene being observed in young tissues early in development. This result is consistent with a previous real-time analysis of the expression pattern of *pPLAIIIδ* [5]. Moreover, our results regarding GUS staining in the pericycle cells of primary and lateral roots indicated a potential function of pPLAIIIδ in the development of lateral roots.

### Altered expression of *pPLAIIIδ* affects plant growth and size in both *Arabidopsis* and *Brassica napus*

**Figure 2:**
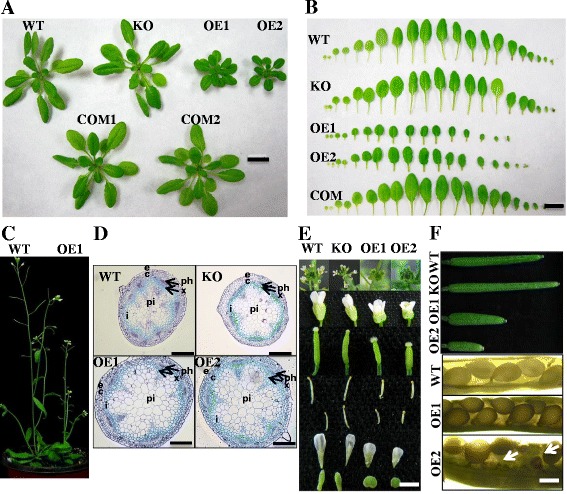
**Altered plant growth and size of knockout and overexpression mutants of** ***pPLAIIIδ*** **. (A)** Morphology of 30-day-old *Arabidopsis* plants. The size of the KO was slightly enlarged, whereas overexpression of *pPLAIIIδ* resulted in smaller plants with more compact leaves and shorter petioles. Bar = 1 cm. **(B)** Individual rosette leaves of 30-day-old plants. The size and the number of rosette leaves of two independent overexpression lines were clearly distinguishable from the WT. From left to right, the leaves were arranged from cotyledons to the youngest rosette leaves. Bar = 1 cm. **(C)** Aerial parts of WT and OE1. WT was clearly taller than OE. **(D)** Cross-sections of the stalks of 6-week-old plants stained with toluidine blue. c, cortex; e, epidermis; i, interfascicular cells; ph, phloem; pi, pith; x, xylem. Bars = 100 μm. **(E)** Morphology of flowers and floral organs. The exposed stigma, smaller flower, altered shape of petal and calyx, and shorter stamen in two OE lines are shown. Inflorescence, flower, stigma, long stamen, short stamen, petal, and calyx are shown from top to bottom. Bar = 3.5 cm. **(F)** Immature siliques of 50-day-old plants and seeds in mature siliques. The siliques of KO plants were slightly longer, whereas those of all OEs were shorter than those of WT plants. The arrangements of seeds in OEs were crowded, and abortions of ovules in OE2 could be observed (arrows). Bar = 100 μm.

To determine the effect of pPLAIIIδ on plant growth and development, an *Arabidopsis* knockout mutant of *pPLAIIIδ* (KO), two independent lines with gain-of-function mutations of *pPLAIIIδ* (OE lines), and the complementary lines of *pPLAIIIδ*-KO (COM) were examined for morphological changes. The KO lines showed no difference in plant size after thirty days of growth in soil compared with the wild-type plants (WT), but the growth of all OE lines was inhibited throughout their lifespan, with fewer and smaller rosette leaves (Figure 2A and B). The 8-week-old OE plants were approximately 25% shorter than the WT and KO plants due to the shortened internodes, resulting in a bushy plant yet with a similar number of cauline leaves to WT (Figure 2C, Table 1). The increase in stem diameter in two OE lines was mainly attributed to the larger pith cells and interfascicular cells, based on histological observations (Figure 2D). OE plants also showed shorter floral organs as well as shorter siliques with more crowded seed arrangement and more aborted ovules (Figure 2E and F, Table 2). Collectively, overexpression of *pPLAIIIδ* inhibited longitudinal growth but promoted transverse expansion in most organs.

**Table 1:** **Morphological measurements of WT, KO, OE1, OE2 and COM** ***Arabidopsis*** **plants**

**Traits**	**WT**	**KO**	**OE1**	**OE2**	**COM**
Plant height (cm, n = 15)	30.77 ± 0.65a	30.41 ± 0.62a	23.04 ± 0.54c	16.31 ± 0.82d	25.54 ± 0.87b
Diameter (mm, n = 15)	1.24 ± 0.04c	1.24 ± 0.03c	1.53 ± 0.03a	1.4 ± 0.04ab	1.29 ± 0.02bc
First internode length (cm, n = 15)	2.37 ± 0.23a	2.55 ± 0.21a	0.85 ± 0.08b	0.65 ± 0.1b	2.32 ± 0.22a
Second internode length (cm, n = 15)	2.37 ± 0.12a	2.44 ± 0.21a	1.00 ± 0.06b	0.67 ± 0.05b	2.00 ± 0.14a
Third internode length (cm, n = 15)	1.87 ± 0.09a	1.88 ± 0.13a	0.71 ± 0.06b	0.42 ± 0.09b	1.7 ± 0.10a
Main inflorescence length (cm, n = 15)	22.19 ± 0.75ab	22.91 ± 0.74a	20.11 ± 0.58ab	14.56 ± 0.83c	19.54 ± 1.07b
First branch length (cm, n = 15)	21.51 ± 0.51a	20.75 ± 0.57a	15.96 ± 0.38b	13.03 ± 0.66c	17.02 ± 0.76b
Number of cauline branches (n = 15)	4.30 ± 0.17A	4.07 ± 0.21A	4.54 ± 0.15A	4.40 ± 0.14A	4.67 ± 0.14A
Number of siliques in main inflorescence (n = 15)	47.54 ± 1.29a	51.31 ± 1.33a	50.86 ± 2.45a	38.69 ± 1.88b	52.58 ± 0.98a
Number of siliques on first branch (n = 15)	25.61 ± 1.06b	28.69 ± 1.30ab	31.87 ± 1.91a	28.17 ± 1.75ab	28.25 ± 0.93ab
Silique length (cm, n = 80)	1.57 ± 0.04a	1.61 ± 0.03a	0.84 ± 0.02c	0.66 ± 0.02d	1.46 ± 0.02b
Fruit pedicel length (cm, n = 80)	0.74 ± 0.02ab	0.72 ± 0.03a	0.38 ± 0.01c	0.28 ± 0.01d	0.57 ± 0.01b
Number of seeds/silique (n = 80)	62.43 ± 0.85a	58.67 ± 1.05a	60.76 ± 1.46a	45.71 ± 1.76b	59.92 ± 0.87a

NOTE: 8-week-old plants were utilized for the morphological analysis; the presented values are means ± SE; mean separation in columns by LSD, uppercase letters indicate P <0.01 and lowercase letters indicate P <0.05.

**Table 2:** **Alternations of the floral organs of** ***pPLAIIIδ*** **KO and OE** ***Arabidopsis*** **plants**

**Floral organ**	**WT**	**KO**	**OE1**	**OE2**
Gynoecia length (mm, n = 20)	2.58 ± 0.07a	2.70 ± 0.05a	2.12 ± 0.06B	1.59 ± 0.05C
Long stamen (mm, n = 20)	2.40 ± 0.03B	2.65 ± 0.03A	1.84 ± 0.03C	1.14 ± 0.03D
Short stamen (mm, n = 20)	1.79 ± 0.03b	1.90 ± 0.05a	1.33 ± 0.05C	0.77 ± 0.03D
Petal length (mm, n = 20)	2.99 ± 0.06B	3.17 ± 0.04A	2.06 ± 0.04C	1.55 ± 0.03D
Calyx length (mm, n = 20)	2.09 ± 0.03B	2.28 ± 0.03A	1.53 ± 0.03C	1.08 ± 0.02D

NOTE: Unfolded floral organs from 6-week-old plants were examined during flowering; the presented values are means ± SE; mean separation in columns by LSD, uppercase letters indicate P <0.01 and lowercase letters indicate P <0.05.

**Figure 3:**
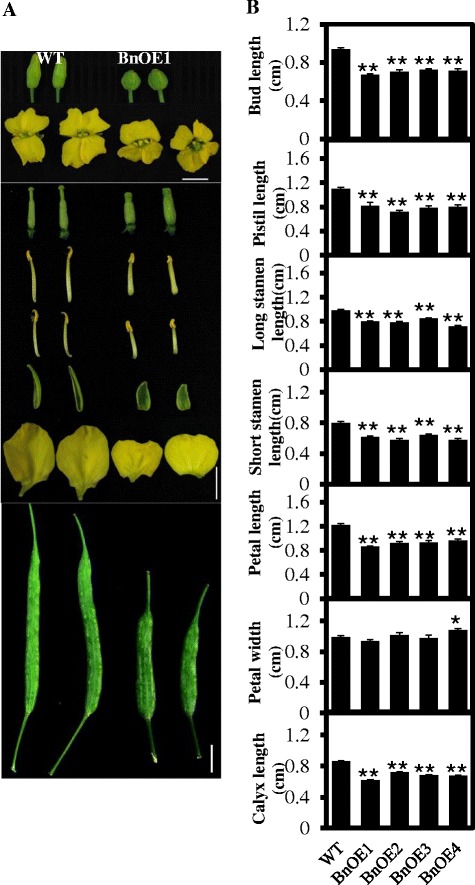
**Altered morphology of buds, flowers, floral organs and siliques of** ***pPLAIIIδ*** **-overexpressing** ***Brassica napus*** **plants. (A)** Morphology of buds, flowers, floral organs, and siliques. Overexpressing *pPLAIIIδ* in *B. napus* leads to morphologic alterations in floral organs and siliques similar to those in *Arabidopsis*. Bar = 1 cm. **(B)** Measurement of buds and various floral organs. Buds and all floral organs in independent lines overexpressing *pPLAIIIδ* (BnOE1 to BnOE4) decrease in length, whereas the width of the petal is increased. All values are means ± SE based on 15 samples. * and ** indicate significant differences at P ≤0.05 and at P ≤0.01, respectively, by Student’s *t* test.

To confirm the effect of pPLAIIIδ on the growth and development of plant organs, we overexpressed *pPLAIIIδ* in J572, a *Brassica napus* cultivar. Four independent transgenic lines (BnOE1 through BnOE4) showed morphological changes similar to those in *Arabidopsis* OE lines (Figure 3 shows changes in the floral organs and siliques), confirming that pPLAIIIδ plays a key role in regulating the growth and development of plant organs.

### Overexpression of *pPLAIIIδ* resulted in defective cell polar growth

**Figure 4:**
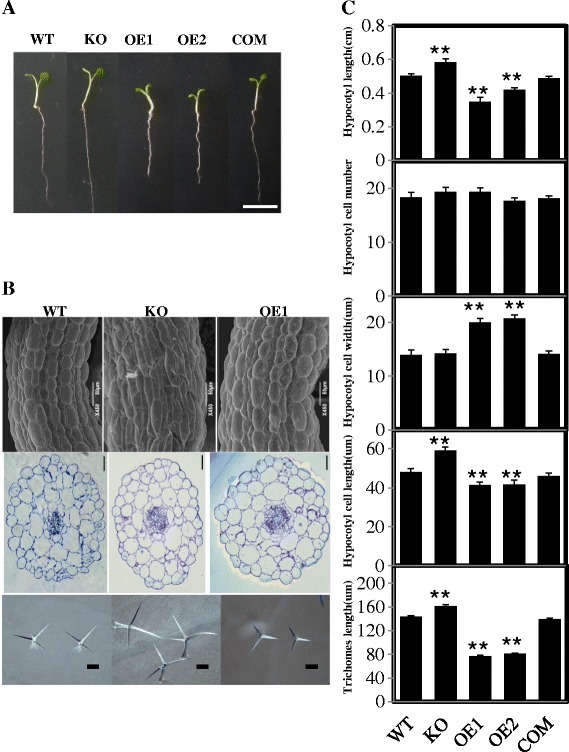
**Morphological and cytological characterisation of hypocotyls and trichomes. (A)** Morphological comparison of the hypocotyls of WT, *pPLAIIIβ*-mutant, KO, OE lines, and COM. Plants were grown in MS medium for 5 d, including WT, *pPLAIIIβ* mutant, KO, OE1, OE2, and COM. Bar = 1 cm. **(B)** Cytological characterisation of the hypocotyl epidermal cells in various lines. Hypocotyl epidermal cells of WT, KO, and OE1 from 6-day-old seedlings showing reduced cell length and increased cell width in the OE plants examined by scanning electron microscopy (first row). Hypocotyl cross-sections of WT, KO, and OE plants stained with aniline blue (second row). KO plants developed longer trichome branches, whereas OE1 plants had shorter branches compared with WT (third row). Bars = 50 μm (first row) or 100 μm (second and third rows). **(C)** The morphological and cytological measurement of hypocotyls and trichomes. Compared with WT, KO plants developed longer hypocotyls with more epidermal cells in the hypocotyl and the trichome branches, whereas OE plants had shorter hypocotyls and trichome branches. There was no difference in cell number on the hypocotyl axis among the genotypes, but OE plants had shorter, wider epidermal cells in the hypocotyl relative to those in WT. All values are the means ± SE of 15 samples. * and ** indicate significant differences at P ≤0.05 and at P ≤0.01, respectively, by Student’s *t* test, compared with WT.

The hypocotyls of *pPLAIIIδ-*KO plants were 15.6% longer, and the hypocotyls of the *pPLAIIIδ-*OE lines were 23.5% shorter relative to those of WT plants (Figure 4C). There was no obvious difference in hypocotyl length between WT and COM (Figure 4B and C). The epidermal and endodermal cells and the cortex cells in OE hypocotyls exhibited increased radial expansion (Figure 4B). The epidermal cell numbers in the hypocotyl WT, KO, OE and COM were similar (approximately 20) (Figure 4C). Moreover, the trichome cell branches were 12.5% longer in KO but 44% shorter in OE compared with WT (Figure 4B and C).

**Figure 5:**
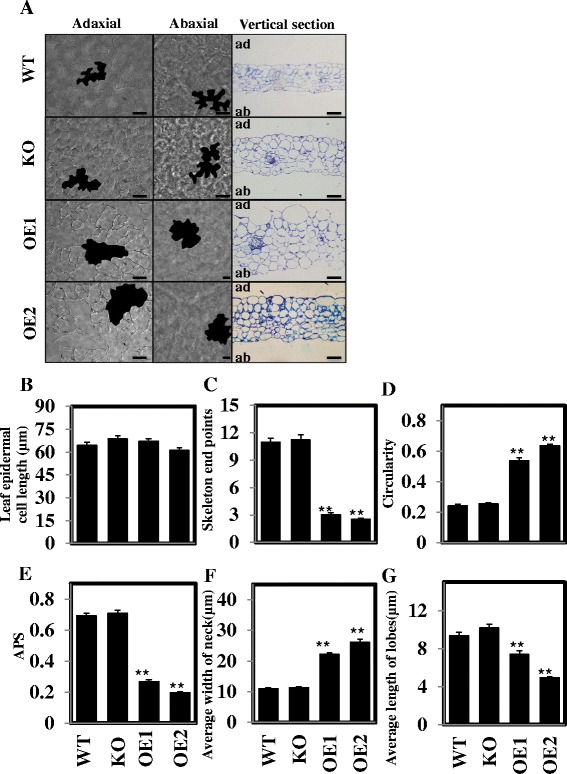
**Overexpression of** ***pPLAIIIδ*** **affect leaf cell development. (A)** Leaf epidermal cells from WT, KO, and OE lines. Reduced convolution of epidermal cells (first and second columns) in OE plants was observed compared with WT and KO. The leaf vertical section of WT, KO OE plants (third column) showed aberrant mesophyll cells that almost completely occupied the spaces in OEs. Bars = 50 μm. **(B-G)** Measurements of cell length, skeleton end points, circularity, APS, neck width, and lobe length of epidermal pavement cells, respectively. All values are means ± SE of ≥15 samples. * and ** indicate significant differences at P ≤0.05 and at P ≤0.01, respectively, by Student’s *t* test, compared with WT. ad, adaxial; ab, abaxial.

A typical interlocking jigsaw-puzzle shape was observed in both WT and KO leaf pavement cells, but the leaf pavement cells of the OE lines developed fewer lobes and indentations, resulting in a less convoluted leaf epidermis (Figure 5A, adaxial and abaxial panels). In the vertical sections of WT and KO leaves, elongated palisade mesophyll cells were packed tightly on the adaxial side; rounded spongy mesophyll cells were packed loosely on the abaxial side. In contrast, all cells in OE plants tended to be circular in shape and organised tightly, resulting in the lack of adaxial-abaxial polarity (Figure 5A, compare the panels in the third column).

Circularity, skeleton end points, and average polarity score (APS) were measured based on the inverse linear relationship of circularity and skeleton end points (see Additional file 2). In WT, KO, and OE plants, no significant difference was shown in the cell length along the longitudinal axis (Figure 5B). However, the skeleton end points in the OE lines (3.01) decreased significantly relative to the WT (10.99) and KO (11.24) lines, whereas the average circularity was higher in the OE plants (0.54) versus the WT (0.24) and KO (0.25) plants (Figure 5C and D). The lower APS (<0.27) of the OE lines compared with those of the WT (0.69) and KO (0.71) lines indicated a defect in the leaf epidermal pavement cell polarity (Figure 5E). The wider necks and shorter lobes of epidermal pavement cells in OE plants suggested that both enhanced radial cell expansion in indentation regions and deficient extension in the lobes contributed to the pavement cell deformation (Figure 5F and G). Taken together, these data indicate that altered *pPLAIIIδ* expression affects the polarity of cell growth.

### Overexpressing *pPLAIIIδ* up-regulates the expression of genes related to ethylene biosynthesis

**Figure 6:**
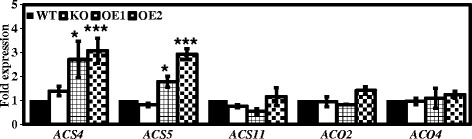
**Overexpression of** ***pPLAIIIδ*** **up-regulates the expression of genes related to ethylene biosynthesis.** The transcript levels of genes involved in ACC biosynthesis were quantified via real-time PCR in WT, OE1, and OE2 plants, using *ACT7* as an internal control. The data are from three biological treatments. Values are means ± SD (n = 3 technical replicates).

As the inhibited hypocotyl elongation observed in OE lines resembles the “triple response” phenotype associated with ethylene [24], we detected the expression of key genes involved in the rate-limiting step of ethylene biosynthesis in *pPLAIIIδ*-OE lines [25]. These genes included five *ACS* (1-aminocyclopropane-1-carboxylate synthase) genes and 2 *ACO* (1-aminocyclopropane-1-carboxylate oxidase) genes, together with *RSA1* (root system architecture 1), which encodes a protein with 1-aminocyclopropane-1-carboxylate synthase activity, and *XBAT32* (XB3 ortholog 2 in *Arabidopsis thaliana*), which mediates the degradation of ACS. Two of the five *ACS*s as well as *RSA1* and *XBAT32* could not be detected in the young seedlings, and *ACO2* and *ACO4* did not show obvious differences among the plant lines (Figure 6). In contrast, the expression of *ACS4* and *ACS5* was up-regulated by 2.5-fold on average in two OE lines compared with WT plants (Figure 6), indicating possible up-regulation of the ethylene biosynthesis pathway in OE plants.

### Altered *pPLAIIIδ* expression changes the endogenous auxin distribution

**Figure 7:**
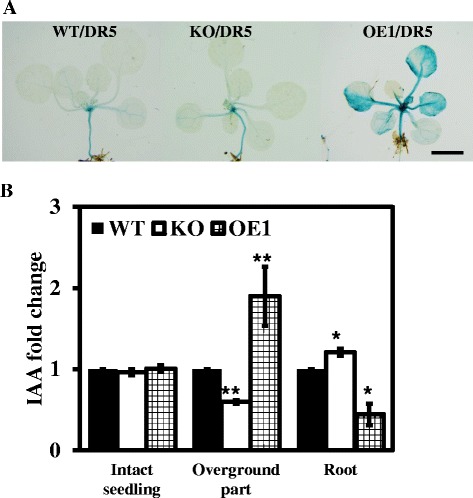
**Altered expression of** ***pPLAIIIδ*** **affects the endogenous auxin distribution. (A)** pPLAIIIδ positively regulates *DR5::GUS* expression. One-week-old seedlings were subjected to GUS staining in the aerial portions of WT, KO and OE1 plants. Bar = 5 mm. **(B)** Changes in free IAA contents in intact seedlings, above-ground tissues and roots among WT, KO and OE1 plants. 7-day-old intact seedlings were grown vertically and collected to measure IAA contents. All values are the means ± SE of 3 biological replicates.

To trace the auxin distribution in lines with altered *pPLAIIIδ* expression, independent *DR5::GUS* plants were generated in WT, KO and OE genetic backgrounds. GUS staining in the hypocotyls did not show significant differences between the WT, KO and OE lines (Figure 7A). However, more intense and widely dispersed GUS staining was observed in the leaves of the OE lines compared with WT and KO plants (Figure 7A), suggesting that the auxin distribution was affected by the enhanced expression of *pPLAIIIδ*.

The free IAA content in intact seedlings was further investigated. There was no difference in the free IAA content observed between WT, KO and OE1 plants when whole seedlings were evaluated (Figure 7B). However, when the free IAA content was examined separately in the above-ground parts of the plants and the roots, the free IAA contents in KO and OE1 plants displayed opposite tendencies in the two tissues compared with WT plants: the above-ground tissues showed a higher free IAA content in OE1 plants and a lower content in KO plants; in contrast, the free IAA content in the roots was decreased in OE1 plants and increased in KO plants (Figure 7B). Taken together, our data indicated that altered *pPLAIIIδ* expression could change the endogenous auxin distribution.

### Response of *pPLAIIIδ* to exogenous IAA induction

**Figure 8:**
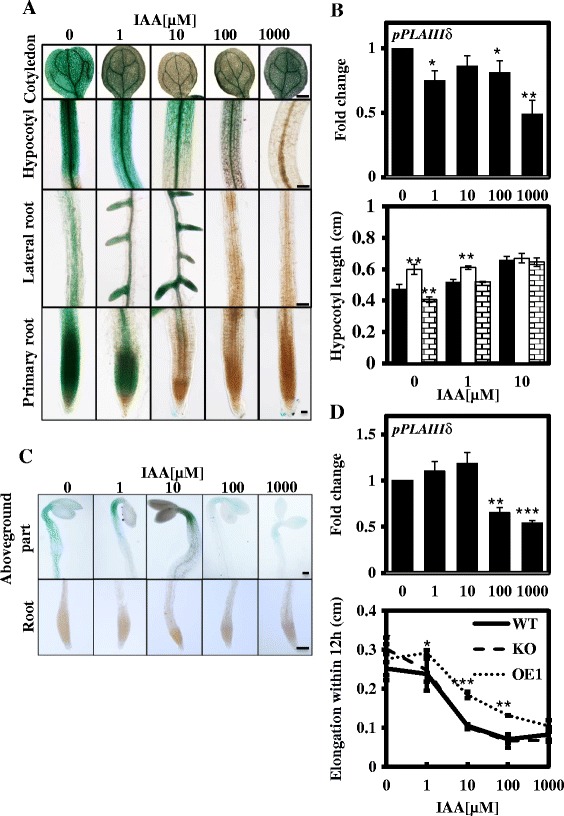
**Response of** ***pPLAIIIδ*** **to exogenous IAA induction. (A)** GUS activity in 7-day-old transgenic *Arabidopsis* plants carrying *pPLAIIIδ:GUS* fusions under treatment with 0 to 1 mM IAA for 48 h. The intensity of GUS staining decreased gradually with increases in the exogenous IAA concentration in multiple organs, except for the sites of the lateral root initiation under 10 μM. Cotyledon, hypocotyl and lateral root, Bar = 500 μm; Primary root, Bar = 100 μm. **(B)** Hypocotyl length of WT, KO and OE1 plants incubated with 0 to 10 μM IAA in the light. The transcript levels of *pPLAIIIδ* in the light were quantified following incubation with 0 to 1000 μM IAA via real-time PCR, using *ACT7* as an internal control. The data are from 3 biological treatments. Values are means ± SD (n = 3 technical replicates). *, ** and *** indicate significant differences at P ≤0.05, P ≤0.01 and P ≤0.001, respectively, by Student’s *t* test. **(C)** GUS activity in 2-d-old dark-grown transgenic *Arabidopsis* plants carrying *pPLAIIIδ:GUS* fusions under treatment with 0 to 1 mM IAA for 12 h. In the dark, GUS activity could not be detected in the roots. In the above-ground parts, the range of GUS staining was restricted with the increase in the IAA concentration. When exogenous IAA was elevated to 100 and 1000 μM, the intensity of GUS staining was markedly decreased. Above-ground parts and roots, bar = 10 μm. **(D)** Hypocotyl elongation in WT, KO and OE1 plants within 12 h under incubation with 0 to 1000 μM IAA in the dark. The transcript levels of *pPLAIIIδ* in the dark were quantified under incubation with 0 to 1000 μM IAA via real-time PCR, using *ACT7* as an internal control. The data are from 3 biological treatments. Values are means ± SD (n = 3 technical replicates).

It has been reported that auxin regulates both the ROP2-actin and the ROP6-MT pathways, resulting in polarised cell growth in the leaf epidermis [26], while the coordination of auxin, ethylene and light controls growth in the hypocotyl [22]. To explore the possible role of pPLAIIIδ in auxin-regulated polarised cell growth, we analysed the promoter sequences of *pPLAIIIδ*. Approximately 25% of the regulatory element motifs were found to be involved in the hormone response, including 4 types of auxin-responsive elements (see Additional files 3 and 4). Such a regulatory pattern in its promoters, together with the auxin responses of *pPLAIIIδ* (see Additional file 5), indicated that the cell deformation in KO and OE lines is likely related to the auxin response of *pPLAIIIδ*. To verify this hypothesis, we examined the GUS activity of transgenic plants harbouring *pPLAIIIδ::GUS* under IAA treatment ranging from 0 to 1000 μM for 48 h. Upon treatment with less than 10 μM IAA, GUS activity was repressed in hypocotyls, cotyledon and primary roots but enhanced in the lateral root initiation zone in a dose-dependent manner (Figure 8A). With the further increase of IAA (>10 μm), *pPLAIIIδ* expression was inhibited in all tissues (Figure 8A). Gene expression analysis via qPCR showed that the transcript levels of *pPLAIIIδ* in the above-ground tissues decreased gradually with the increase of exogenous IAA in the light (Figure 8B).

To verify the role of auxin in the hypocotyl morphogenesis of KO and OE lines, we compared the hypocotyl elongation rate among WT, KO and OE plants under different exogenous IAA treatments (from 0 to 10 μM) in the light. Hypocotyl elongation was enhanced in all WT, KO and OE plants by exogenous IAA treatment, but the sensitivity of the hypocotyl elongation in response to exogenous IAA stimulus varied among these lines (Figure 8B). Compared with the control (0 μM IAA), the hypocotyl lengths of KO, WT and OE plants under 1 μM IAA treatment increased by 1.7%, 9.5% and 28.1%, respectively; the hypocotyl lengths of KO, WT and OE1 under 10 μM IAA treatment increased by 11.7%, 39.2% and 59%, respectively (Figure 8B).

To rule out the possible impact of light on the auxin response, we compared hypocotyl elongation in 2-d-old dark-grown seedlings responding to different IAA treatments in the dark over 12 h. GUS activity could not be detected in the roots; in the above-ground parts, the range of *pPLAIIIδ* expression was restricted in response to the lower IAA concentration treatments (1 and 10 μM), and the higher concentrations of exogenous IAA (100 and 1000 μM) suppressed the expression of *pPLAIIIδ* significantly (Figure 8C). Gene expression analysis through qPCR confirmed the GUS staining results: the expression of *pPLAIIIδ* was not markedly influenced by the 1 μM and 10 μM IAA treatments, whereas the transcript levels of *pPLAIIIδ* were decreased by 50% under the high IAA concentration treatments (100 μM and 1000 μM) (Figure 8D). The above results demonstrated that the hypocotyl elongation of WT and KO plants was suppressed under treatment with exogenous IAA, and this inhibition was especially obvious in KO plants under 1 μM IAA treatment (Figure 8D). Taken together, these data indicated that the differences in hypocotyl length observed between KO, WT and OE1 plants gradually diminished with further increases in the IAA concentration from 1 to 10 μM in the light, and the differential hypocotyl elongation rates recorded among the various plant lines were reversed by treatment with 1 μM IAA in the dark.

**Figure 9:**
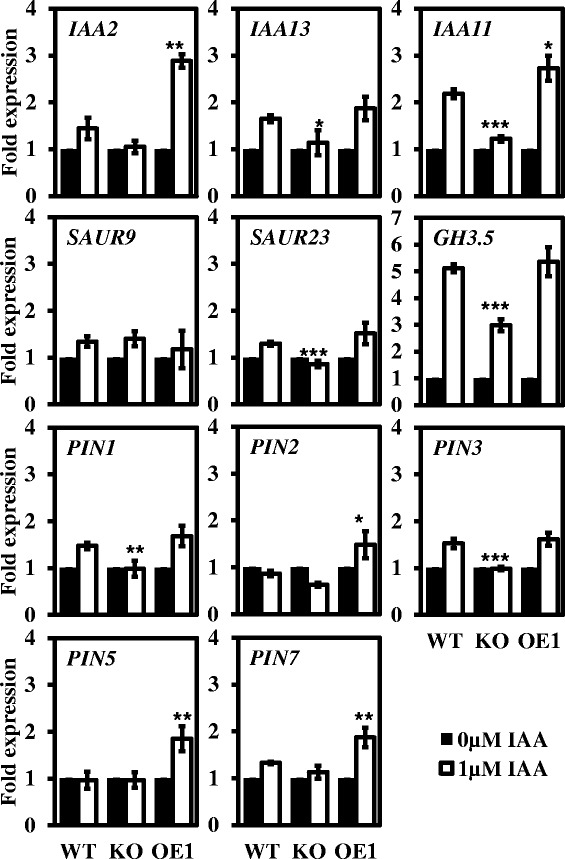
**Expression of auxin-inducible genes in dark-grown plant lines in response to auxin treatments.** The transcript levels of early auxin-responsive genes and auxin efflux carrier genes in WT, KO and OE1 were detected after treatment with 1 μM auxin for 12 h in the dark. The data were normalized based on the transcript levels of the genes in the corresponding non-treated samples. The data are from three biological treatments. Values are means ± SD (n = 3 technical replicates). *, ** and *** indicate significant differences at P ≤0.05, P ≤0.01 and P ≤0.001, respectively, by Student’s *t* test.

Next, we monitored the auxin response in the different plant lines by analysing the expression of auxin-activated genes under treatment with 1 μM IAA in the dark. Three groups of auxin-responsive genes, the *IAA* genes, *SAUR* genes and *PIN* genes, as well as *GH3.5*, were selected to examine the different auxin responses among WT, KO and OE1 plants. Under treatment with 1 μM IAA, the transcript levels of the *SAUR* genes and *GH3.5* did not display obvious changes, while three *IAA* genes showed a weakened auxin response in KO plants and an enhanced auxin response in OE1 plants compared with WT plants, with 3-fold higher expression of *IAA2* being detected. A similar trend was found in the expression of five PIN genes among various plant lines: under treatment with 1 μM IAA in the dark, the transcript levels of *PIN5* and *PIN7* in OE1 plants were elevated 2-fold, while there was no significant difference in WT and KO (Figure 9).

**Figure 10:**
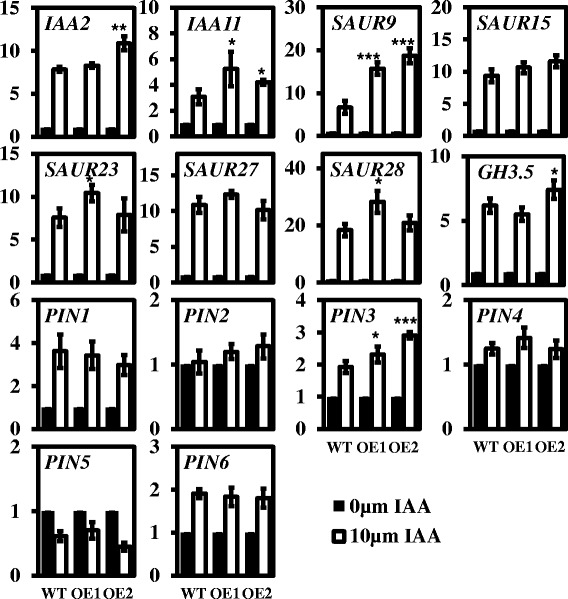
**Expression of auxin-induced genes in IAA-treated WT and OE seedlings.** The transcript levels of early auxin-responsive genes and auxin efflux carrier genes in response to treatment with 10 μM IAA for 30 min in the light were quantified via real-time PCR in WT, OE1 and OE2 plants, using *ACT7* as an internal control. The data were normalised based on the transcript levels of the genes in the corresponding non-treated samples. The data are from three biological treatments. Values are means ± SD (n = 3 technical replicates). *, ** and *** indicate significant differences at P ≤0.05, P ≤0.01 and P ≤0.001, respectively, by Student’s *t* test.

Labusch et al. detected a weakened auxin response in a *pPLAIIIδ* loss-of-function mutant, but the only auxin-sensitivity phenotype observed was for root growth [8]. To further verify the enhanced auxin response of OE lines, we investigated the expression of the early auxin-inducible genes responding to treatment with 10 μM IAA for 30 min in light-grown seedlings. Among the 8 examined early auxin-inducible genes, *IAA2*, *IAA11*, *SAUR9*, *SAUR23*, *SAUR28*, and *GH3.5* were significantly up-regulated in OE1 and/or OE2 plants (Figure 10). *IAA2*, *IAA11*, *SAUR9*, and *GH3.5* were previously reported to respond weakly to an auxin stimulus in a *pPLAIIIδ* mutant [8]. The *SAUR*9 gene exhibited 17-fold up-regulation on average compared with 6-fold up-regulation in the corresponding WT plants (Figure 10).

To determine the changes in auxin polar transport under exogenous auxin treatment, we further analysed the transcript levels of *PIN* genes. Consistent with previous studies, *PIN1* and *PIN3* were up-regulated, while *PIN5* was down-regulated significantly, by 60% under treatment with10 μM IAA within 30 min. The transcript level of *PIN6* was also increased, by approximately 2-fold. Compared with WT plants, the transcript levels of *PIN3* increased significantly in two OE lines (Figure 10), suggesting that the change in the auxin response induced by altered *pPLAIIIδ* expression may result in disturbance of auxin polar transport. These data therefore clearly suggested that pPLAIIIδ positively regulates the auxin response.

### Altered *pPLAIIIδ* expression modified PA content significantly

**Figure 11:**
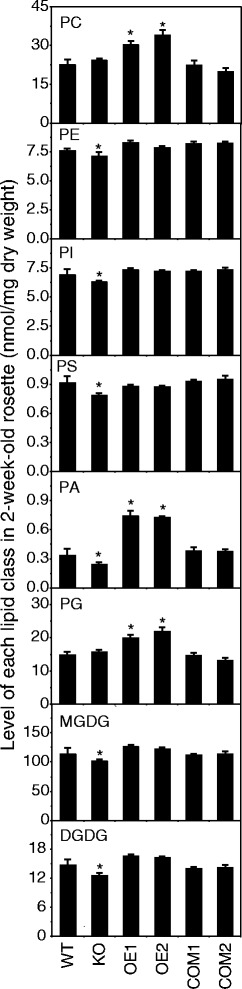
**Effect of altered** ***pPLAIIIδ*** **expression on lipid content.** Lipid molecular species of phospholipids and galactolipids in WT, KO, OE, and COM lines. Phospholipids include PC, PE, PI, PS, PA, and PG; galactolipids include MGDG and DGDG. Values are means ± SE (n = 5); each replicate contained at least 3 plant rosettes. * Significant at P <0.05 compared with the WT based on Student’s *t* test. PC, phosphatidylcholine; PE, phosphatidylethanolamine; PI, phosphatidylinositol; PS, phosphatidylserine; PA, phosphatidic acid; PG, phosphatidylglycerol; MGDG, monogalactosyldiacylglycerol; DGDG, digalactosyldiacylglycerol.

To better understand the effect of pPLAIIIδ on the cellular lipidome, we profiled the classes of membrane phospholipids and galactolipids. The major cellular phospholipids profiled include PC, phosphatidylethanolamine (PE), phosphatidylinositol (PI), phosphatidylserine (PS), phosphatidic acid (PA), and phosphatidylglycerol (PG). The levels of PE, PI, PS, and PA were 6%, 9%, 14%, and 27% lower in the leaves of the KO mutant compared with WT (Figure 11). The levels of total PC, PA, and PG were 35%, 118%, and 35% higher in the OE mutant leaves compared with WT. The major cellular lipids in chloroplasts are monogalactosyldiacylglycerol (MGDG) and digalactosyldiacylglycerol (DGDG). The levels of MGDG and DGDG were 10% and 14% lower in KO mutants compared with WT (Figure 11). The levels of molecular species of these cellular lipids tended to be lower in KO mutants and higher in OE mutants, in which the PA content was the molecular species most affected by altered *pPLAIIIδ* expression.

PA is mainly produced through the PLD- or phospholipase C (PLC)-diacylglycerol kinase (DGK) pathways. To identify the potential mechanism regulating the PA contents of plants with altered *pPLAIIIδ* expression, we analysed the expression of *PLD* and *PLC* genes. Among the 12 *PLD* genes examined, 11 did not show a notable change on transcript levels among the WT, KO and OE plant lines, and *PLDβ2* could not be detected in the seedlings. The 12 *PLC* genes detected included all 9 phosphoinositide-specific phospholipase genes (*PI-PLC*) and 3 non-specific phospholipase genes (*NPC*). Although lower expression of 9 *PI-PLCs* was observed in KO plants, while higher expression of these genes was detected in OE lines, the difference was not statistically significant (see Additional file 6). Thus, we ruled out the possibility that PLD- or PLC-DGK pathways may affected by altered *pPLAIIIδ* expression at the transcriptional level to produce more PA among the mutants.

## Discussion

### Altered *pPLAIIIδ* expression results in distinct auxin-responsive phenotypes

Our phenotypic, cytological, and molecular analyses of the growth and development of KO and OE plants provide strong evidence for the involvement of pPLAIIIδ in cell morphogenesis (Figures 4 and 5). The observation of similar morphological changes following altered *pPLAIIIδ* expression in *pPLAIIIβ* mutants [5] indicated that these gene present redundant functions to some extent. These morphological changes resemble auxin-regulated modifications in different organs recorded in previous studies, including changes in the siliques [27], internodes [28], hypocotyls [11,29], floral organs [30] and leaf shape [31,32]. The pleiotropic effects of auxin on plant growth and development can be attributed to the regulation of both cell division and/or cell morphogenesis by auxin [33]. Considering the dominant role of auxin in hypocotyl morphogenesis [29], we compared the numbers of cells along the hypocotyl epidermis among WT, KO, OE and COM plants. All of the plant lines exhibited approximately 20 cells along the long axis of the hypocotyl (Figure 4C), which is consistent with previous studies [34]. Based on this result, it is unlikely that the distinct phenotypes of the hypocotyls of KO and OE plants can be attributed to cell division. Moreover, the deficient polarised cell morphogenesis that was observed in the hypocotyl epidermis cells of KO and OE provided solid evidence that altering *pPLAIIIδ* expression induced modifications in polarised cell morphology, resulting in the phenotype observed in the hypocotyls of KO and OE plants (Figure 4A and B).

Other researchers have reported suppressed growth in the primary roots and an elevated lateral root density in response to NAA stimuli in *pPLAIIIδ* loss-of-function mutants [8]. In the present study, when *pPLAIIIδ* expression was observed with GUS staining, *pPLAIIIδ* was detected predominantly in the pericycle cells of primary root tissue (Figure 1F), which have been reported to dedifferentiate and proliferate to form the lateral root primordium during the initiation and development of lateral roots under auxin regulation [35,36]. Additionally, the exogenous IAA treatments (1 and 10 μM) not only suppressed GUS staining in the primary roots but also altered its distribution, with intensive GUS activity appearing in the lateral root primordium (Figure 8A), indicating that pPLAIIIδ is involved in auxin-regulated lateral root development.

### pPLAIIIδ is involved in the auxin-dependent polarised cell growth

Inhibited hypocotyl elongation is a diagnostic sign of an ethylene response, redirecting the longitudinal growth of the hypocotyl in a radial direction, resembling what is observed in light-grown OE plants [24] (Figure 4). Our real-time analysis showed that the expression of the *ACS4 and ACS5* genes in young tissues was significantly up-regulated in OE seedlings (Figure 6). It has been demonstrated that the expression of *ACS4* and *ACS5* is positively related to ethylene production [25,37]. Enhancement of ACC biosynthesis has been indicated to occur in *pPLAIIIδ*-OE lines. We further showed that exogenous ethephon treatment did not affect the expression of *pPLAIIIδ* significantly and that the differential hypocotyl elongation rates of KO and OE plants were not diminished by ethephon treatment (see Additional file 7). Thus, the effect of pPLAIIIδ on hypocotyl elongation may not be entirely attributed to the enhanced production of ethylene.

Hypocotyl photomorphogenesis have been associated with the actions of auxin and ethylene to a large extent in previous studies [22]. Auxin can stimulate the expression of ACC synthase genes [38,39]. Considering the different effects of auxin and ethephon treatments on the hypocotyl elongation rates of KO and OE plants, we inferred that auxin is more likely to contribute to the suppression of hypocotyl elongation as well as the activation of ACC biosynthesis in OE plants. In the present study, the total free IAA concentrations detected among intact WT, KO and OE1 seedlings did not show any significant differences, indicating a lack of influence on auxin biosynthesis in these plants (Figure 7B). However, intensive GUS staining and higher free IAA contents were detected in the above-ground tissues of OE lines compared with WT and KO plants (Figure 7A and B), indicating a significant alteration of the auxin distribution in OE plants. Taken together, these data indicated that the suppression of hypocotyl elongation in OE plants was related to the alteration of auxin transport, rather than auxin biosynthesis.

Our data also showed that altered *pPLAIIIδ* expression affects the polarity of leaf pavement cells (Figure 5). It has been reported that two mutually antagonistic signalling pathways, the ROP2-actin and ROP6-MT pathways, regulate the development of leaf pavement cells and that defects in the two pathways lead to deficient polarity during leaf pavement cell growth [20,31,32,40], similar to the phenotype of OE plants (Figure 5). Recently, it was reported that auxin regulates the ROP2-actin and ROP6-MT pathways through ABP1 (auxin-binding protein 1) to participate in the polarised growth of leaf pavement cells, which depends on the formation of a basipetally increasing longitudinal gradient of free IAA along the lamina [11]. At the base of the leaf, the relatively higher free IAA level accompanied by a lower level of ABP1 exhibited a lower auxin affinity, promoting cell division. In contrast, the relatively lower free IAA level accompanied by higher *ABP1* expression indicated a higher auxin affinity at the tip of the leaf and resulted in polarised cell growth. ABP1, as an auxin receptor, perceives a uniform concentration of auxin to activate the antagonistic ROP2-actin and ROP6-MT pathways, guiding the formation of lobes and indentations, respectively in leaf pavement cells at different sites [26,41]. In the present study, the results of *DR5::GUS* staining and the measurement of free IAA contents revealed a significant increase in the free IAA concentration in above-ground tissues, confirming the disturbed auxin distribution (Figure 7A and B). Given the similarity between the deficient polar growth of leaf pavement cells observed in OE plants in the present study and in the *ABP1* mutant [12], it is likely that the abnormal auxin distribution in the leaf pavement cells of OE plants disturbs the formation of the basipetally increasing longitudinal gradient of free IAA along the lamina, consequently leading to the interdigitation defect.

### *pPLAIIIδ* is involved in the regulation of the auxin response in plants

Our results revealed auxin-responsive GUS activity in *pPLAIIIδ::GUS*-transformants in both the light and dark (Figure 8A and C) as well as the types of auxin-responsive elements in the *pPLAIIIδ* promoter (see Additional files 3 and 4), indicating that *pPLAIIIδ* is likely an auxin-responsive gene. Our gene expression analysis showed a slight auxin-response expression of *pPLAIIIδ* (Figure 8B and D), consistent with the previous report [8]. Under 1 and 10 μM IAA treatments, the expression of *pPLAIIIδ* was induced in the lateral roots but repressed in primary roots (Figure 8A). Above data suggested that the exogenous IAA stimuli might regulate the spatial expression pattern, and the counteraction of the differential auxin-responsive expression of *pPLAIIIδ* in various tissues might be the reason that the overall transcriptional level of *pPLAIIIδ* was influenced by IAA treatments slightly.

On the other hand, the distinct auxin sensitivities detected during hypocotyl elongation among WT, KO, and OE plants and the differential gene expression observed in response to the exogenous IAA stimuli in both the light and dark (Figure 8B and D) provided evidence that *pPLAIIIδ* plays a positive role in regulating the auxin response. The majority of the auxin response genes regulate the auxin signalling loop itself, and the direct changes at the transcriptional level are therefore difficult to detect, including those in the *GH3s, IAA* and *PIN* genes [8]. However, the differential auxin response among the various plant lines was amplified under the stress condition. In the dark, the expression of *IAA2* and *IAA11* was lower in KO plants and higher in OE1 plants, presenting a significant response to treatment with 1 μM IAA (Figure 9). Although there was no direct evidence for the involvement of the two genes in hypocotyl elongation, it has been found that several *IAA* genes affect the development of the hypocotyl. For example, *axr2-1*(*IAA7*) plants display a shorter hypocotyl in the dark [42]; the reduced auxin sensitivity of *shy2-1*(*IAA3*) plants inhibits hypocotyl elongation [43]; *shy1-1*(*IAA6*) plants exhibit suppressed hypocotyl elongation in the dark [44]; and *iaa18-1*(*IAA18*) plants show an increase in hypocotyl length [45]. Moreover, the twofold up-regulation of *PIN5* and *PIN7* detected in OE1 plants compared with WT and KO plants (Figure 9) also reflected the different intracellular auxin concentrations among the WT, KO and OE1 lines. Thus, the differential expression of *IAA* genes as well as the consequent change in the intracellular auxin concentration among WT, KO and OE1 plants is likely responsible for the different hypocotyl elongation rates of the various plant lines responding to IAA treatments in the dark. In addition, it has been shown that the differential expression of auxin-induced genes is attenuated within 3 h [8]. However, in dark-grown OE1 seedlings, *IAA2, IAA11, PIN5* and *PIN7* maintained higher auxin responses after IAA treatment for 12 h, confirming that the enhanced auxin response and transport induced by overexpressing *pPLAIIIδ* could remain for quite some time.

Previous researches demonstrated a delay in the up-regulation of auxin-induced gene expression in a *pPLAIIIδ* loss-of-function mutant [8]. Our finding of significant up-regulation of auxin-induced gene expression in *pPLAIIIδ*-overexpressing plants under treatment with 10 μM IAA for 30 min in the light further confirmed that pPLAIIIδ is positively involved in the auxin response and transport (Figure 10). Among the identified up-regulated genes, *SAUR9* was up-regulated by 17-fold on average in OE lines, compared with 6-fold up-regulation in WT plants (Figure 10). Most of the *SAUR* genes can mediate auxin-induced cell elongation during the morphogenesis of multiple organs [46,47]. For example, overexpression of the *SAUR32* gene results in apical hook opening and a shorter hypocotyl in *Arabidopsis* [48], and overexpression of *OsSAUR39* affects not only shoot, but also root morphology, the effects of which can be restored by exogenous auxin in rice [49]. Additionally, consistent with previous findings in *pPLAIIIδ* mutants, overexpression of *pPLAIIIδ* also affected the response of *PIN3* to IAA stimuli (Figure 10). PIN3 plays a positive role in cell elongation in the hypocotyl [22,50], resulting from the important lateral auxin efflux carrier PIN3 guiding the auxin flow toward the epidermal cell layers to control growth [51]. Hence, the higher expression of *SAUR9* and *PIN3* observed within 30 min in response to IAA treatment in the light not only indicated a stronger auxin response, but also may contribute to auxin hypersensitivity during hypocotyl elongation in OE plant lines.

### pPLAIIIδ regulated the auxin response via PA

Among the products generated from pPLAIIIδ-catalysed reactions, PA might be partially responsible for the change in the auxin response detected in plants with altered *pPLAIIIδ* expression. Li et al. performed a pharmacological experiment (FM4-64 treatment) to show that PLDζ2 positively regulates the auxin response through one of its products, PA [52]. In this study, we observed a 27% decrease in the PA in KO lines and a 118% increase in the OE lines (Figure 11), suggesting that the altered auxin responses might be attributed to the changed PA content in plants from KO and OE lines. Consistent with the broader distribution of auxin in the OE plants (Figure 7A), the expanded GUS staining region detected following PA treatment in the roots of *DR5::GUS* seedlings revealed enhancement of the auxin response, which was attributed to faster PIN2 cycling [52]. PA is essential for vesicle trafficking during PIN cycling to facilitate early endosome fusion with the plasma membrane and the stimulate actin polymerisation [52,53]. Abnormal PIN cycling would cause multiple auxin-related defects [54-56]. All of these findings support a model in which pPLAIIIδ acts through PA to positively regulate the auxin response.

In vivo, PA is generated either directly via PLD pathways or indirectly via PLC-DGK pathways [7]. Alternatively, the two types of pathways would be regulated at the post-transcriptional level or at the transcriptional level [57-61]. Our gene expression analysis of the transcript levels of *PLD* and *PLC* genes did not show significant variation (see Additional file 6), excluding the possibility that the altered PA contents of the KO and OE plant lines resulted from transcriptional regulation of the PLD and/or PLC-DGK pathways. Regarding post-transcriptional regulation, the hydrolysed products of pPLAIIIδ (free fatty acids and lysophospholipids) have been shown to function in the activation of PLD, resulting in the production of PA [62,63]. Therefore, the question of how PLD- and/or PLC-DGK-dependent pathways are activated in KO and OE plant lines deserves further study.

Based on the above analyses, we propose a model for the involvement of *pPLAIIIδ* in auxin-regulated polarised cell growth: overexpressing *pPLAIIIδ* induced an increase in the PA content and, consequently, led to enhancement of the auxin response. In OE plants without IAA treatment, the endogenous enhanced auxin response activated ethylene biosynthesis and affected the auxin distribution, causing deficient cell polarity in the hypocotyl and leaf epidermis; under IAA treatment, the up-regulation of early auxin-responsive genes and enhanced auxin transport counteracted the suppressive effect of endogenous ethylene on hypocotyl elongation and promoted increased hypocotyl growth.

## Conclusions

This study demonstrated that *pPLAIIIδ* was involved in auxin-responsive polarised cell growth, acting through PA, resulting in deficient organ development in *Arabidopsis* and *B. napus*. Although the members of the patatin-related phospholipase subfamily III (pPLAIIIs) have been implicated in the auxin response, it remains unclear whether and how these genes affect plant and cell morphogenesis. Until now, the understanding of the biological functions of patatin-related phospholipase subfamily III has been limited. Exploring the mechanism of pPLAIIIδ in regulating auxin-responsive cell morphogenesis should provide insights not only into the biological function of pPLAIIIδ but also into the roles of the phospholipase-dependent signal transduction networks in auxin-responsive polarised cell growth.

## Methods

### Plant materials

The isolation of a homozygous T-DNA insertion mutant for *pPLAIIIδ* (*pPLAIIIδ*-KO), as well as the generation of the complementation lines (*pPLAIIIδ*-*COM*) and overexpression lines (*pPLAIIIδ*-*OE*) for *pPLAIIIδ* were reported previously [64]. The plasmid for the *pPLAIIIδ* overexpression vector was also transferred into *Brassica napus* cv. J572 according to the protocol described by Zhou et al. [65]. The transgenic plants were screened and confirmed by PCR. More than 10 independent transgenic lines were obtained, and 4 of them (BnOE1 through BnOE4) were selected for phenotypic assays.

To clone the promoter region of *pPLAIIIδ*, the genomic sequence of *pPLAIIIδ* from the promoter region to the coding sequence was isolated by PCR from Col-0 *Arabidopsis* genomic DNA using the two primers listed in Additional file 8. The cloned promoter was fused into the binary vector pMDC163 containing the *uidA* gene for plant transformation. The transgenic plants were screened and confirmed by PCR, and 4 independent homozygous transgenic lines from T3 generations were used for GUS assays.

### Plant growth and treatments

Surface-sterilised seeds were plated on 0.5× Murashige and Skoog salt agar. After stratification at 4°C for 2 d in the dark, seedlings were grown vertically on plates in a growth room with a 16-h-light/8-h-dark cycle, at 22/21°C, under cool fluorescent white light (200 μmol m^−2^ s^−1^). For the experiments on soil-grown plants, the plants were grown in growth chambers with a 16-h-light/8-h-dark, at 22/20°C, 50% humidity, at 200 μmol m^−2^ s^−1^ of light intensity. For hormone treatments, 3-day-old seedlings were transferred to plates with different concentrations of IAA (0, 1 μM, 10 μM, 100 μM and 1 mM) for 48 h, and 7-day-old seedlings were transferred to plates with 10 μM IAA for different lengths of time.

### Histochemical GUS activity

To assay GUS activity, dissected samples were incubated with 5-bromo-4-chloro-3-indolyl-β-D-glucuronide (X-Gluc) solution as described by Hemerly et al. [66]. The seedlings were incubated at 37°C in the dark for 12 h. X-Gluc-treated samples were rinsed with 95% (v/v) ethanol and transferred to 70% (v/v) ethanol. The samples were observed with a Nikon ECLIPSE 80i compound microscope.

### Cytology

Hypocotyls from 7-day-old plants and the 3^rd^ and 4^th^ unfolded leaves from 30-day-old plants (≥15 samples) were fixed in FAA (3.7% formaldehyde, 5% glacial acetic acid, 50% ethanol) for 4 h under a vacuum and decoloured with 75% ethanol. The leaves were further hyalinised in Hoyer’s solution containing 10:1:1.5:2.5 of chloral hydrate:glycerin:Arabic gum:ddH_2_O for 2 h. Images were obtained using a Nikon ECLIPSE 80i differential interference contrast microscope with a Nikon-DS-Ri1 CCD camera and measured with the Image J software. The length, width, and number of cells in the epidermal hypocotyl single-cell file and the length of the trichomes from 4 zones adjacent to the central main vein in the leaves were measured. The central zones of leaves with ≥5 intact cells with clear outlines were selected for the pavement cell analyses. The perimeter and area were used to calculate the circularity of leaf pavement cells (circularity = 4π area/(perimeter)^2^). The cell traces mentioned above were filled, copied and pasted into a new file for the skeleton end-point count representing the lobe number, and the skeletonize plugin of the Image J software was run to process the binary image after thresholding [67].

For scanning electron microscopy, fresh samples were fixed in FAA containing 1% Triton X-100, dehydrated through a graded ethanol series, and dried using a Hitachi HCP-2 critical point dryer (Hitachi, Japan). The samples were then mounted on scanning electron microscopy stubs, sputter-coated with gold using an Eiko IB-5 ion coater (Eiko Engineering Company, Ibaraki, Japan), and then observed under a JSM-3690/LV scanning electron microscope (Jeol, Japan).

For semi-thin section analyses, samples were fixed in FAA for 4 h under vacuum conditions, dehydrated in an ethanol series (30, 50, 70, 85, 95, 100, and 100%) for 1 h for each step, and then immersed in Technovit 7100 resin (Heraeus Kulzer, Wehrheim, Germany) following the manufacturer’s protocol. Semi-thin (2 μm) sections were made using a Leica Ultracut R ultra-microtome (Leica Microsystems, Wetzlar, Germany) and stained with toluidine blue (0.5% toluidine blue and 0.2 M sodium citrate buffer, pH 4.5) for 30 s. Images were obtained using a Nikon ECLIPSE 80i compound microscope.

### Lipid profiling

Lipids were extracted from 2-week-old soil-grown rosettes and analysed by electrospray ionisation-tandem mass spectrometry (ESI-MS/MS), and the lipids were profiled as previously described [68,69].

### RNA extraction and Real-Time PCR

Total RNA samples were prepared from various tissues using TRIzol reagent (Invitrogen, USA) and treated with DNase I (Fermentas, USA) according to the manufacturer’s instructions. For each sample, 2 μg of RNA was converted to cDNA with the Thermo Scientific RevertAid First Strand cDNA Synthesis Kit (Fermentas). Gene-specific primers were designed using online software (http://www.idtdna.com/scitools/Applications/RealTimePCR). The primer sequences are listed in Additional file 8. Real-time PCR was performed using the TransStart Top Green qPCR SuperMix kit (TransGen, China) as previously described [70] and using the Bio-Rad CFX96 Real-Time system (Bio-Rad). Relative quantification was performed using the comparative cycle threshold method, and the relative amount of PCR product amplified using the designed primer sets was normalised to the control gene *ACT7*. The data are expressed as mean ± SD (n =3 technical replicates).

### HPLC-MS/MS analyses

Fresh plant tissues (200 to 600 mg) from 7-day-old *Arabidopsis* seedlings were weighted precisely, then frozen and ground to a powder in liquid Nitrogen. The IAA fraction was dissolved in 1 mL of 80% methanol/H_2_O and extracted at 4°C for 24 hours. Then, the samples were centrifuged for 10 min at 4°C, and the supernatant was transferred to a fresh tube. The extraction was quickly repeated using 300 μl of 80% methanol/H_2_O for 1 hour, and the mixture of the two extracts was dried with a nitrogen evaporator (Organomation Associates Incorporated, USA). The IAA fraction was redissolved in 300 μl of 80% methanol/H_2_O and injected into an high-performance liquid chromatography-tandem mass spectrometry (HPLC-MS/MS) (America Agilent Technologies, USA). The region of the external standard used to normalize the data covered 0, 0.05, 0.1, 0.2, 0.4, 0.8 and 1.6 ng/mL IAA. The protocol was based on the descriptions of Sugawara et al. [71] and Xiangqing Pan et al. [72] with some modifications.

## Availability of supporting data

The data about the temporal expression and the root expression pattern of *pPLAIIIδ* are available to download at the Genevestigator website (http://www.genevestigator.com/). The data about the time-course of *pPLAIIIδ* expression in response to 1 μM IAA treatment are available from the AtGenExpress Visualization Tool website (http://jsp.weigelworld.org/expviz/expviz.jsp?experiment=development&normalization=absolute&probesetcsv=At3g63200&action=Run).

## Additional files

Additional file 1: Figure S1. Expression pattern of *pPLAIIIδ.*
Additional file 2: Figure S2. Linear relationship between circularity and skeleton end-points in leaf pavement cells. Additional file 3: Figure S3. Bioinformatics analysis of promoter elements in *pPLAIIIδ.*
Additional file 4: Table S1. Hormone-related elements in the promoter of *pPLAIIIδ.*
Additional file 5: Figure S4. Time-course of *pPLAIIIδ* expression in response to 1 μM IAA treatment based on data from the website. Additional file 6: Figure S5. Expression of genes involved in PLD- and PLC-DGK pathways. Additional file 7: Figure S6. Response of *pPLAIIIδ* to ethephon. Additional file 8: Table S2. Primers for mutant identify, molecular cloning and real-time PCR in quantitative measurement of transcript levels.

## Abbreviations

pPLAIII: Patatin-related phospholipase subfamily III
PIN: Auxin efflux carrier PIN-FORMED family
LRR: Leu-rich repeat motif
LPE: Lysophoshatidylethanomine
LPC: Lysophosphatidylcholine
LPA: Lysophosphatidic acid
PLD: Phospholipase D family
STURDY: A gain-of-function mutant of *pPLAIIIδ*
AUX1/LAX: AUX1/LIKE-AUXIN proteins
MDR/PGP: ATP-dependent multi-drug resistance/P-glycoprotein-type ABC transporters
KO: An *Arabidopsis* T-DNA insertion mutant of *pPLAIIIδ*
OE lines: Two independent lines of gain-of-function mutant of *pPLAIIIδ*
COM: Complementary lines of *pPLAIIIδ*-KO
WT: Wild-type plants
BnOE1 through BnOE4: Four independent *Brassica napus* transgenic lines of *pPLAIIIδ*
APS: Average polarity score
ACS: 1-aminocyclopropane-1-carboxylate synthase
ACO: 1-aminocyclopropane-1-carboxylate oxidase
RSA1: Root system architecture 1 protein
XBAT32: XB3 ortholog 2 in *Arabidopsis thaliana*
MT: Microtube
IAA: Indoleacetic acid
PC: Phosphatidylcholine
PE: Phosphatidylethanolamine
PI: Phosphatidylinositol
PS: Phosphatidylserine
PA: Phosphatidic acid
PG: Phosphatidylglycerol
MGDG: Monogalactosyldiacylglycerol
DGDG: Digalactosyldiacylglycerol
PI-PLC: Phosphoinositide-specific phospholipase
NPC: Non-specific phospholipase
ABP1: Auxin-binding protein 1
DGK: Diacylglycerol kinase
X-Gluc: 5-bromo-4-chloro-3-indolyl-β-D-glucuronide
ESI-MS/MS: Electrospray ionization-tandem mass spectrometry
HPLC-MS/MS: High-performance liquid chromatography-tandem mass spectrometry
