# Supplementary material for: Patatin-related phospholipase pPLAIIIδ influences auxin-responsive cell morphology and organ size in Arabidopsis and Brassica napus
Source: BMC Plant Biol. 2014 Nov 27;14:332. doi: 10.1186/s12870-014-0332-1 (PMC4253999; doi:10.1186/s12870-014-0332-1)
Supplement: Additional file 1: Figure S1. — Expression pattern of pPLAIIIδ. [file 12870_2014_332_MOESM1_ESM.pdf]

Supplemental Figure S1

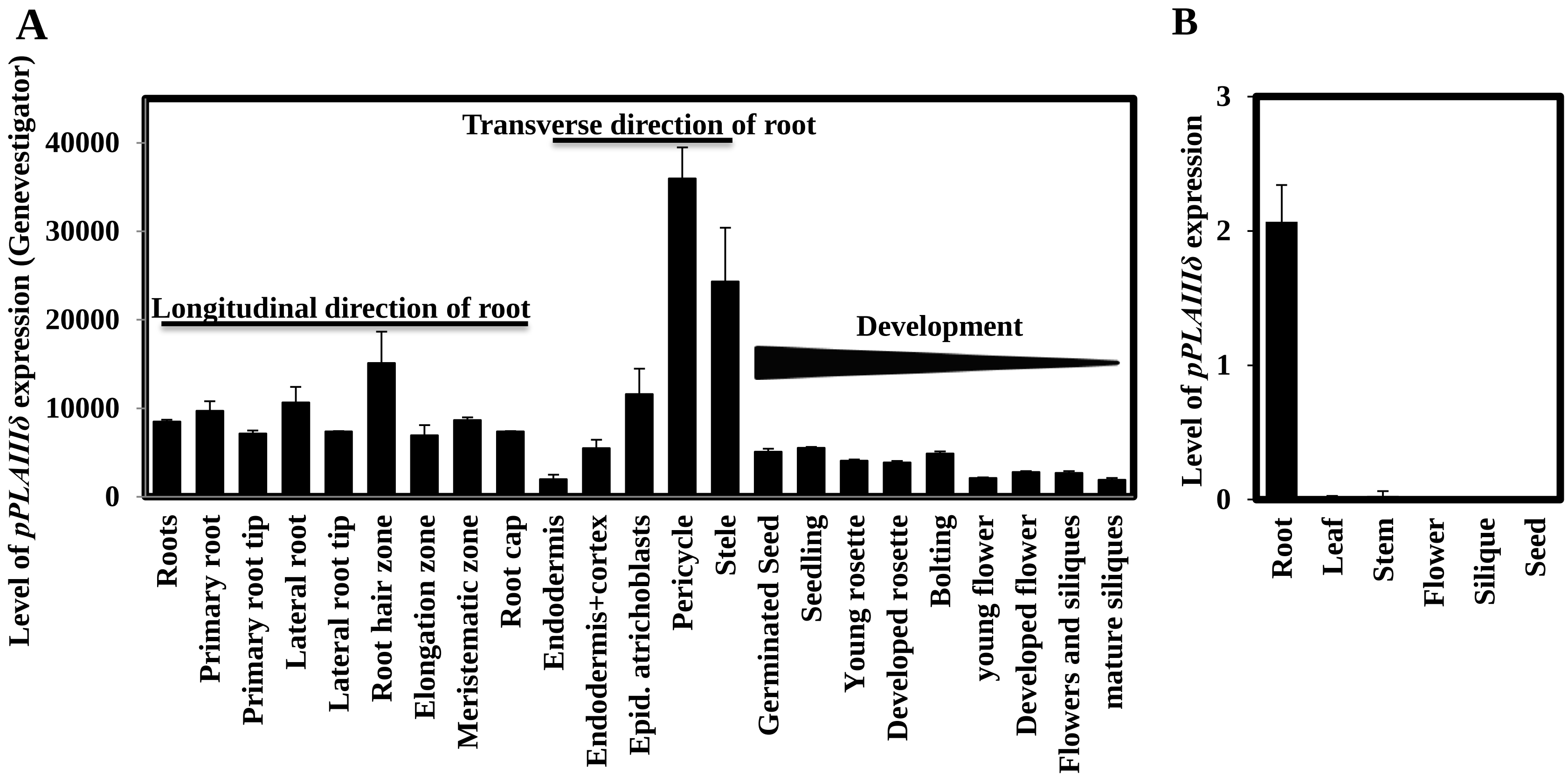

Supplemental Figure S1. Expression pattern of *pPLAIIIδ*

(A)The temporal expression of *pPLAIIIδ* and the root expression pattern in longitudinal and transverse directions based on data from Genevestigator (http://www.genevestigator.com).

(B)The relative expression ratios of *pPLAIIIδ* in various *Arabidopsis* tissues quantified by real-time PCR normalized to *ACT7*. Values are means ± SD (n=3 replicates).
