# Supplementary material for: Patatin-related phospholipase pPLAIIIδ influences auxin-responsive cell morphology and organ size in Arabidopsis and Brassica napus
Source: BMC Plant Biol. 2014 Nov 27;14:332. doi: 10.1186/s12870-014-0332-1 (PMC4253999; doi:10.1186/s12870-014-0332-1)
Supplement: Additional file 2: Figure S2. — Linear relationship between circularity and skeleton end-points in leaf pavement cells. [file 12870_2014_332_MOESM2_ESM.pdf]

Supplemental Figure S2

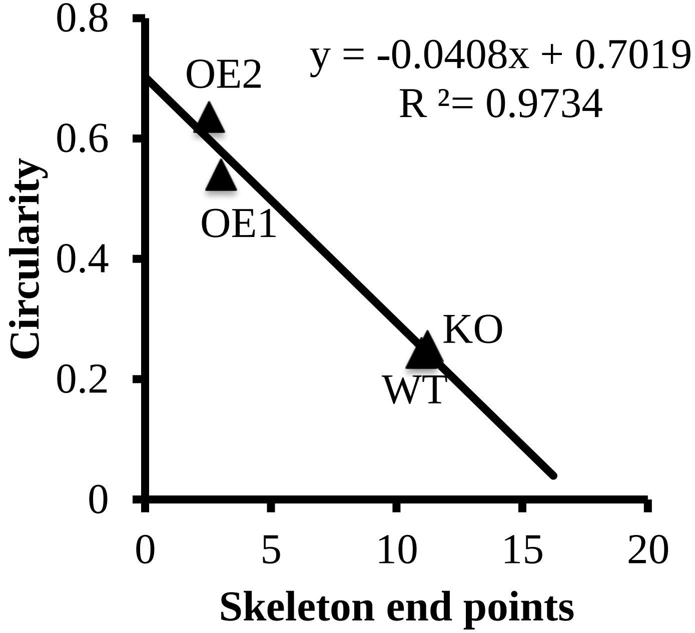

Supplemental Figure S2. Linear Relations between Circularity and Skeleton End-Points of the Leaf Pavement Cell.

The circularity and skeleton end-points for pavement cell shape in WT, KO, OE1 and OE2 leaves exhibited an inverse linear relation.
