# Supplementary material for: Patatin-related phospholipase pPLAIIIδ influences auxin-responsive cell morphology and organ size in Arabidopsis and Brassica napus
Source: BMC Plant Biol. 2014 Nov 27;14:332. doi: 10.1186/s12870-014-0332-1 (PMC4253999; doi:10.1186/s12870-014-0332-1)
Supplement: Additional file 3: Figure S3. — Bioinformatics analysis of promoter elements in pPLAIIIδ. [file 12870_2014_332_MOESM3_ESM.pdf]

Supplemental Figure S3.

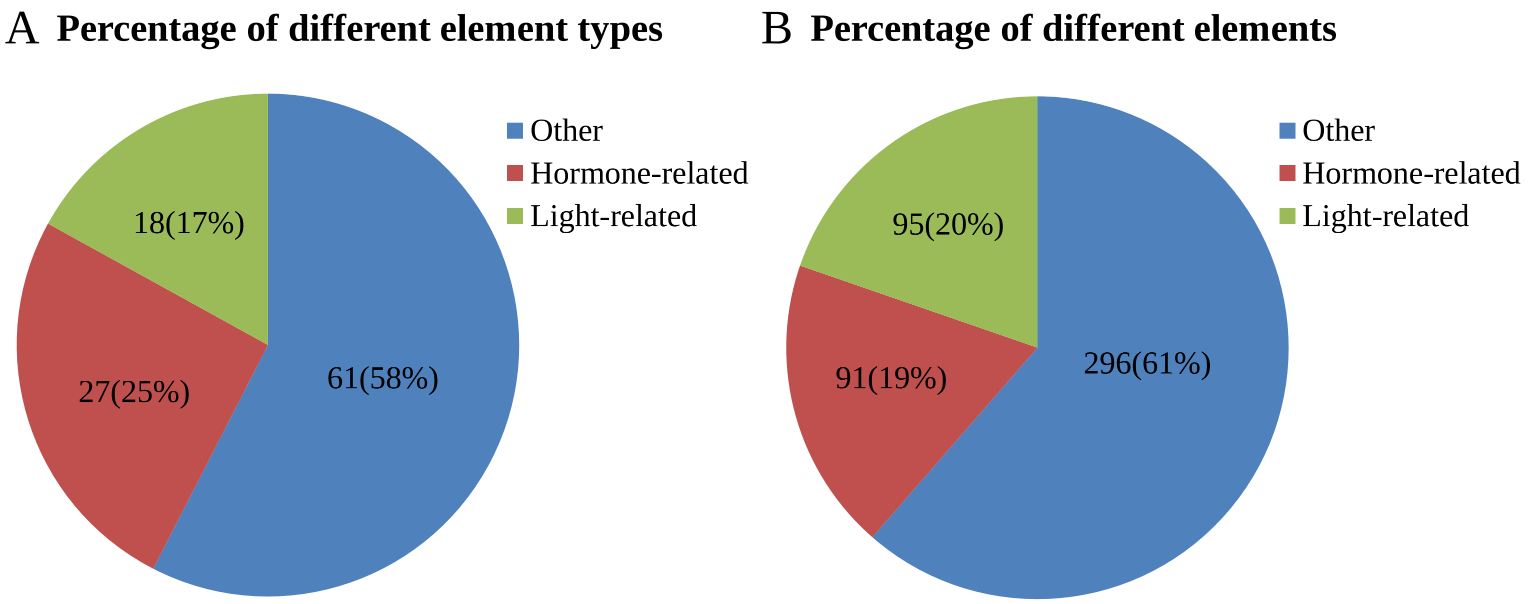

Supplemental Figure S3. Bioinformatic analysis of promoter elements in *pPLAIIIδ*.
(A) Types of *pPLAIIIδ* promoter elements related to various hormones. The hormone-related and light-related elements accounted for 25% and 17% of total elements, respectively.
(B) The promoter of *pPLAIIIδ* included 19% hormone-response sites and 20% the light-response sites.
