# Supplementary material for: Patatin-related phospholipase pPLAIIIδ influences auxin-responsive cell morphology and organ size in Arabidopsis and Brassica napus
Source: BMC Plant Biol. 2014 Nov 27;14:332. doi: 10.1186/s12870-014-0332-1 (PMC4253999; doi:10.1186/s12870-014-0332-1)
Supplement: Additional file 4: Table S1. — Hormone-related elements in the promoter of pPLAIIIδ. [file 12870_2014_332_MOESM4_ESM.pdf]

Table S1 Hormone-relative elements in the promoter of *pPLAIIIδ*

Site name	Site(strand)	Element
ABRERATCAL	646 (+) MACGYGB	ABRE 2
AGCBOXNPGLB	628 (+) AGCCGCC	Ethylene-responsive element
ABRELATERD1	647 (+) ACGTG	ABRE 3
ASF1MOTIFCAMV	1005 (+) TGACG	Auxin and/or salicylic acid 4
CPBCSPOR	1555(+)TATTAG	Cytokinin-enhanced Protein Binding 2
DPBFCOREDCDC3	1278(+)ACACNNG	ABA-responsive 3
GAREAT	1036 (-) TAACAAR	GA/GARE
LECPLEACS2	1154 (+) TAAAATAT	Ethylene-responsive element
MYBGAHV	1036 (-) TAACAAA	GA
MYCCONSENSUSAT	201 (+) CANNTG	ABA 4
PYRIMIDINEBOXHVEPB1	717 (+) TTTTTTCC	GA/ABA/GARE 2
PYRIMIDINEBOXOSRAMY1A	1061 (+) CCTTTT	GA 3
TATCCAOSAMY	473(+)TATCCA	Gibberellin Response 2
WBOXATNPR1	348(+)TTGAC	Salicylic acid (SA)-induced 9
WRKY71OS	26 (+) TGAC	GA 14
SURECOREATSULTR11	81 (+) GAGAC	ARF centrol cis 3
ARFAT	282(-)TGTCTC	ARF (auxin response factor) binding site
ARR1AT	543(+)NGATT	Cytokinin-regulated transcription 28
CACGCAATGMGH3	414(+)CACGCAAT	Auxin inducibility
CACGTGMOTIF	1279(+)CACGTG	Abscisic acid-induced transcription 2
CAREOSREP1	47(-)CAACTC	Gibberellin-upregulated
ELRECOREPCRP1	1251(+)TTGACC	Salicylic acid-induced
GCCCORE	629(+)GCCGCC	Ethylene-responsive element
MYB1AT	108(+)WAACCA	Abscisic acid signaling 2
MYB2CONSENSUSAT	352(+)YAACKG	Abscisic acid signaling
MYBCORE	352(-)CNGTTR	Flavonoid biosynthesis
MYBCOREATCYCB1	353(+)AACGG	Cell cycle phase-independent activation
