# Supplementary material for: Patatin-related phospholipase pPLAIIIδ influences auxin-responsive cell morphology and organ size in Arabidopsis and Brassica napus
Source: BMC Plant Biol. 2014 Nov 27;14:332. doi: 10.1186/s12870-014-0332-1 (PMC4253999; doi:10.1186/s12870-014-0332-1)
Supplement: Additional file 5: Figure S4. — Time-course of pPLAIIIδ expression in response to 1 μM IAA treatment based on data from the website. [file 12870_2014_332_MOESM5_ESM.pdf]

Supplemental Figure S4

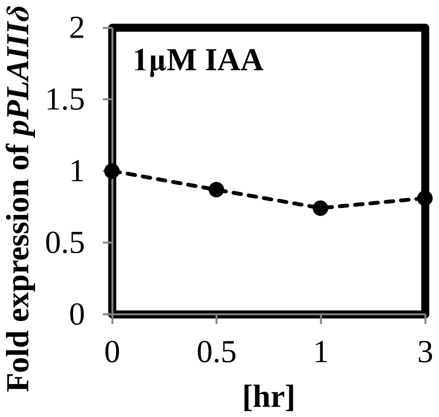

Supplemental Figure S4. Time course of *pPLAIIIδ* expression responding to auxin treatment based on data from website.

(http://jsp.weigelworld.org/expviz/expviz.jsp?experiment=development&normalization=absolute&probesetcsv=At3g63200&action=Run).

Under 1 μM IAA treatment, the expression of *pPLAIIIδ* was only down-regulated slightly.
