# Supplementary material for: Patatin-related phospholipase pPLAIIIδ influences auxin-responsive cell morphology and organ size in Arabidopsis and Brassica napus
Source: BMC Plant Biol. 2014 Nov 27;14:332. doi: 10.1186/s12870-014-0332-1 (PMC4253999; doi:10.1186/s12870-014-0332-1)
Supplement: Additional file 6: Figure S5. — Expression of genes involved in PLD- and PLC-DGK pathways. [file 12870_2014_332_MOESM6_ESM.pdf]

Supplemental Figure S5

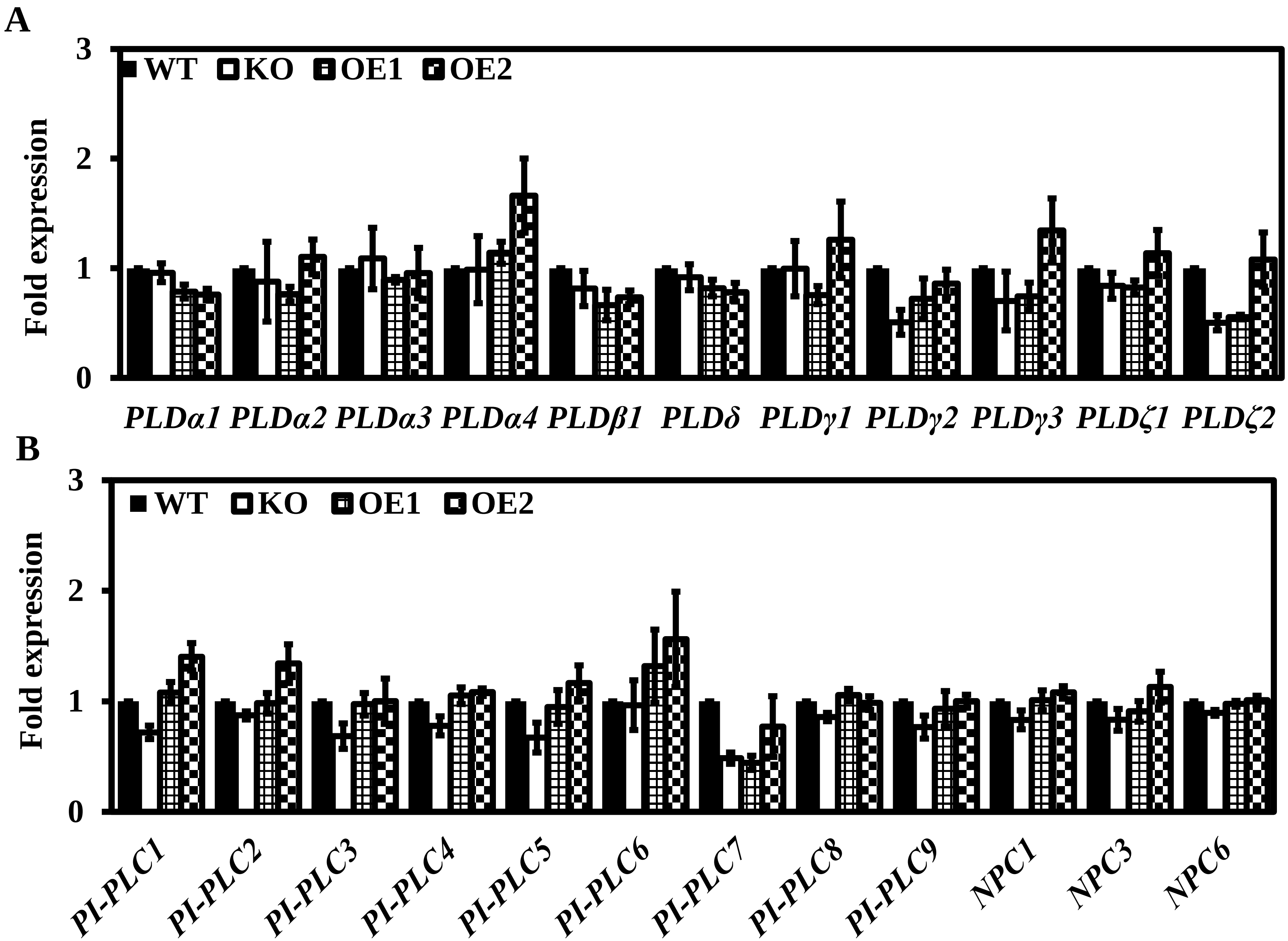

Supplemental Figure S5. Expression of genes involved in PLD-and PLD-DGK-pathways
(A) The expression of *PLD* genes involved in PLD-dependent PA production. Except *PLDβ2*, 11 of 12 *PLD* genes could be detected in the young seedlings.
(B)The expression of *PLC* genes involved in PLC-DGK-dependent PA production.
Real-time PCR data were normalized to *ACT7*. The data came from three biological treatments. Values are means ± SD (n=3 technical replicates).
