# Supplementary material for: Patatin-related phospholipase pPLAIIIδ influences auxin-responsive cell morphology and organ size in Arabidopsis and Brassica napus
Source: BMC Plant Biol. 2014 Nov 27;14:332. doi: 10.1186/s12870-014-0332-1 (PMC4253999; doi:10.1186/s12870-014-0332-1)
Supplement: Additional file 7: Figure S6. — Response of pPLAIIIδ to ethephon. [file 12870_2014_332_MOESM7_ESM.pdf]

Supplemental Figure S6.

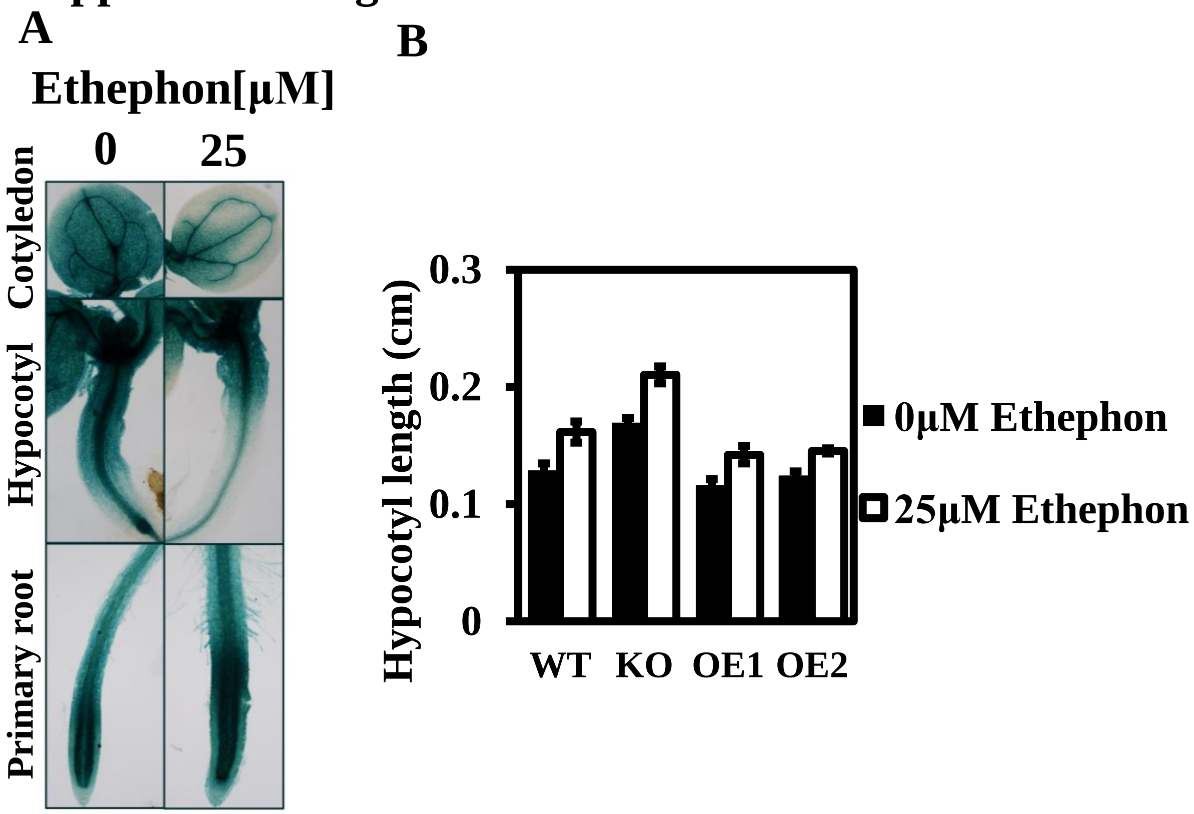

Supplemental Figure S6. Response of *pPLAIIIδ* to Ethephon.

(A)5-old-day plants harboring *pPLAIIIδ:GUS* were treated with 25µM Ethephon for 48 h, and GUS activity was detected as indicated by staining intensity in cotyledon, hypocotyl and primary root.

(B) Hypocotyl length of WT, KO, OE1 and OE2 with 25µM Ethephon incubation in the light. 3-old-day plants were tansferred to the medium supplemented 25µM Ethephon for 48h.
