# Supplementary material for: Patatin-related phospholipase pPLAIIIδ influences auxin-responsive cell morphology and organ size in Arabidopsis and Brassica napus
Source: BMC Plant Biol. 2014 Nov 27;14:332. doi: 10.1186/s12870-014-0332-1 (PMC4253999; doi:10.1186/s12870-014-0332-1)
Supplement: Additional file 8: Table S2. — Primers for mutant identify, molecular cloning and real-time PCR in quantitative measurement of transcript levels. [file 12870_2014_332_MOESM8_ESM.pdf]

Table S2 Primers for mutant identify, molecular cloning and real-time PCR in quantitative measurement of transcript levels.

Gene	AGI	Description	Primers
pPLAIIIδ	AT3G63200	Screening for T-DNA insertional mutant of Salk_029470C	Forward 5'-CAAGCAACAAATATTAGCTGCCCAAAC-3'
			Reverse 5'-TCGCAGTGAGAGAGCCATTTCT-3'
			T-DNA detective primer 5'-ATTTTGCCGATTTCGGAAC-3'
pPLAIIIδ	AT3G63200	Cloning *pPLAIIIδ* gene for overexpression lines	Forward 5'-ATTTAATTAAATGGAGATGGATCTCAGCAAGGTT-3'
			Reverse 5'-ATGGCGCGCCAACGGCCGTCAGCGAGAGGGTTAA-3'
pPLAIIIδ	AT3G63200	Cloning intact *pPLAIIIδ* gene including native promoter region for complementation line	Forward 5'-AGGCGCGCCAAACTATCTCGTGTCGC-3'
			Reverse 5'- AGGCGCGCCACTCTGTGCTGGCTATC-3'
pPLAIIIδ	AT3G63200	Cloning the promoter of *pPLAIIIδ* for the expression pattern analysis	Forward 5'-GGGATTAATTAAGTGTCGTGGGAGCAGTGGA-3'
			Reverse 5'- AAACTCTAGAACGGCCGTCAGCGAGAG-3'
pPLAIIIδ	AT3G63200	Patatin-like protein 9 (PLP9)	Forward 5'-TCAAGCCGTTCAGTGTAGTG-3'
			Reverse 5'-AGTCATCTACGCCGTTTACTG-3'
ACS4	AT2G22810	Encodes 1-aminocyclopropane-4-carboxylate synthase	Forward 5'-GGTTGGGAAGAGTACGAGAAG-3'
			Reverse 5'-GCTGCGTCTGTGTTTTGTG-3'
ACS5	AT5G65800	Encodes 1-aminocyclopropane-4-carboxylate synthase	Forward 5'- TCACGAATCCATCTAACCCAC -3'
			Reverse 5'-CCCGAACATAGTGCCTGAATAG -3'
ACS11	AT3G49700	Encodes 1-aminocyclopropane-4-carboxylate synthase	Forward 5'-GGTTTGTTCTGTTGGGTTGAC-3'
			Reverse 5'-AATGACACGATGAGCCTGG-3'
ACO2	AT1G62380	Encodes 1-aminocyclopropane-1-carboxylic oxidase	Forward 5'-CGGGAAGTATAAGAGTGTGCTG-3'
			Reverse 5'-GGGTACTCGGAATCTTTCTCG-3'
ACO4	AT1G05010	Encodes 1-aminocyclopropane-1-carboxylic oxidase	Forward 5'-TTTCTACCTCAAGCACCTTCC-3'
			Reverse 5'-CGGCGAAGTCTTTCATTAACG-3'
IAA2	AT3G23030	Early auxin-responsive gene	Forward 5'-CCAGTGAGATCTTCCCGTAAG-5'
			Reverse 5'-CGATCTTGCGAAGGTAAGGAG-5
IAA11	AT4G28640	Early auxin-responsive gene	Forward 5'-TGAGCTTGGTCTTACGTTGAG-6'
			Reverse 5'-TCCTCTTGATCCCAGCAATTAC-6
IAA13	AT2G33310	Early auxin-responsive gene	Forward 5'-AACTCGGTGAAAAGGCTACG-3'
			Reverse 5'-CTCTCGTTTGGTTCTTGATTTCG-3'

SAUR9	AT4G36110	Early auxin-responsive gene	Forward 5’-GAGGTGCTCAAGTCTAGGAAAG-3’
			Reverse 5’-CACGAGATTGGGACCACATAG-3’
SURA15	AT4G38850	Early auxin-responsive gene	Forward 5’-AGGAGTTTCTTGGGTGCTAAG-5’
			Reverse 5’-CATAGACCGCCATGAATCCTC-5
SURA23	AT5G18060	Early auxin-responsive gene	Forward 5’-TTTCTTGCAGTGTACGTAGGAG-3’
			Reverse 5’-ATCGTTAAGCCACCCATCG-3
SURA27	AT3G03840	Early auxin-responsive gene	Forward 5’-AACGAGCAAAGCACCAAAAG-5’
			Reverse 5’-CGAGAAGTTACAGTGAGGAAGG-4
SURA28	AT3G03830	Early auxin-responsive gene	Forward 5’-AACGAGCAAAGCACCAAAAG-4’
			Reverse 5’-AGAAGGGCCTGAAATGAAGG-4
GH3.5	AT4G27260	Early auxin-responsive gene	Forward 5’-CTCGAAAACGGCAAAGGAATG-7’
			Reverse 5’-GTTGGTGTAAGGATCATAGGGTC-7
PIN1	AT1G73590	PIN-FORMED 1	Forward 5’-TCCATGTTGCCATTATCCAGG-6’
			Reverse 5’-ACATCCCAAATATCACCGCAG-6
PIN2	AT5G57090	PIN-FORMED 2	Forward 5’-CCGTAAGGTTCTTGACTGGAC-7’
			Reverse 5’-TGGCGAAAACAAAAGGAACG-7
PIN3	AT1G70940	PIN-FORMED 3	Forward 5’-TGGTGATTTACTGCGTGTCG-5’
			Reverse 5’-GTACTCCTTCGCAAACACAAAG-5
PIN4	AT2G01420	PIN-FORMED 4	Forward 5’-AACCCAAACACGTACTCCAG-3’
			Reverse 5’-ACATAGCCATTCCAAGACCAG-3
PIN5	AT5G16530	PIN-FORMED 5	Forward 5’-CAAGGTAATCGAAGCAATGGTTC-3’
			Reverse 5’-ACTCAATGGTGAAGAGCGG-3
PIN6	AT1G77110	PIN-FORMED 6	Forward 5’-GCCTCATACTAACAGTCGTGG-3’
			Reverse 5’-GTGACCAGACAAGACCTAAGAG-3
PIN7	AT1G23080	PIN-FORMED 7	Forward 5’-ATTACGTGGAGACCTATTGCG-4’
			Reverse 5’-TGTACTCAAGATTGCGGGATG-4
PI-PLC1	AT5G58670	Phosphatidylinositol-specific phospholipase C1	Forward 5’-GGTGGATGTGGCTATGTGAA-3’
			Reverse 5’-GTCCATATTCCATCCTTCTCCAG-3
PI-PLC2	AT3G08510	Phosphatidylinositol-specific phospholipase C2	Forward 5’-TGACCAATATTCACCGCCTG-3’

			Reverse 5’-ACTCAAACACCTCATCCCAAG-3
PI-PLC3	AT4G38530	Phosphatidylinositol-specific phospholipase C3	Forward 5’-GAGTGAAGTCCAAGGAGAACG-3’
			Reverse 5’-GAGAGTTGGTATCGCTGAAGAG-3
PI-PLC4	AT5G58700	Phosphatidylinositol-specific phospholipase C4	Forward 5’-TCTGCTCCGTGTTGAAGTTC-3’
			Reverse 5’-TCCCTTGCGATTAAAGAGTGG-3
PI-PLC5	AT5G58690	Phosphatidylinositol-specific phospholipase C5	Forward 5’-CACTTGGTCCTCTCCTAACTTC-3’
			Reverse 5’-TCCGGTACTGTTAGCTGAAAC-3
PI-PLC6	AT2G40116	Phosphatidylinositol-specific phospholipase C6	Forward 5’-GGGATATGGGAAATCTCTGTGG-3’
			Reverse 5’-AGGGTCAAAGACTTCATCGTG-3
PI-PLC7	AT3G55940	Phosphatidylinositol-specific phospholipase C7	Forward 5’-TGTATTTGACCCGGAAGCTAC-3’
			Reverse 5’-GCAATCCCGACCCTTGTATAG-3
PI-PLC8	AT3G47290	Phosphatidylinositol-specific phospholipase C8	Forward 5’-ATTAGTATTGCCGGTGTACCC-3’
			Reverse 5’-CAAGGTCAGGATAGGTCAAAGG-3
PI-PLC9	AT3G47220	Phosphatidylinositol-specific phospholipase C9	Forward 5’-TTAGGATCAGTATTGCCGGTG-3’
			Reverse 5’-CAAGGTCAGGATAGGTCAAGG-3
NPC2	AT3G03520	Non-specific phospholipase C2	Forward 5’-AGGAGCTTAACCCAGAAATCG-3’
			Reverse 5’-CGGGTCAGATGTAGAAAGTGG-3
NPC3	AT1G07230	Non-specific phospholipase C3	Forward 5’-GACCATCTCCTAAACTCATATCCAG-3’
			Reverse 5’-AGTGTTTTCGTCTGCTCCTG-3
NPC6	AT3G48610	Non-specific phospholipase C6	Forward 5’-AACAAGTATTCGGGTCTGGG-3’
			Reverse 5’-AGCCTTTCATTACAGTCTCCG-3
PLDα1	AT3G15730	Phospholipase Dα1	Forward 5’-CCCCTTCTCCTGTTATGTTCC-4’
			Reverse 5’-TCTTTCCCACTTACAAGCCC-4
PLDα2	AT1G52570	Phospholipase Dα2	Forward 5’-ACACTCCGTTTCACTCCTTG-8’
			Reverse 5’-AGCAATAGGTCCTTCAAGACG-8
PLDα3	AT5G25370	Phospholipase Dα3	Forward 5’-CTTTCACCAGCCGAACTTTG-14’
			Reverse 5’-ACCTTGTTTCATCCACCTCTG-14
PLDα4	AT1G55180	Phospholipase Dα4	Forward 5’-GTGGGAGATTTACAGTGGAGAC-9’
			Reverse 5’-ACACTAATTGGATAGGCGACC-9

*PLD*β1	AT2G42010	Phospholipase Dβ1	Forward 5'-TGGAAAGCGACATAGTTGGG-5'<br>Reverse 5'-ATCACATAGATCAAGCCCACC-5
*PLD*β2	AT4G00240	Phospholipase Dβ2	Forward 5'-CGACCCAACTTCACAAAACATC-12'<br>Reverse 5'-ACCCAACTATGTCGCTTTCC-12
*PLD*γ1	AT4G11850	Phospholipase Dγ1	Forward 5'-ATAGACATGGAAAGTGCTGGG-15'<br>Reverse 5'-CAACTCCCCTAATGTACCTTCG-15
*PLD*γ2	AT4G11830	Phospholipase Dγ2	Forward 5'-AAGGTGCTATGTTGAGTCTGTC-6'<br>Reverse 5'-CTCAAAGGGAAGTATGTACCGG-6
*PLD*γ3	AT4G11840	Phospholipase Dγ3	Forward 5'-AAGGTGCTGTGTTGAGTCTG-13'<br>Reverse 5'-CTTAAAGGGAAGTATGTACCGGG-13
*PLD*δ	AT4G35790	Phospholipase Dδ	Forward 5'-AGGACGCTCATGTTATGGAC-7'<br>Reverse 5'-TCTTCCCAACACTTCCCATG-7
*PLD*ζ1	AT3G16785	Phospholipase Dζ1	Forward 5'-TGCCGCTGTCAGATTATACG-11'<br>Reverse 5'-AGAGAACGGTAAGCAGAATGG-11
*PLD*ζ2	AT3G05630	Phospholipase Dζ2	Forward 5'-ATCTCTGTTTTGGGCGGTAC-10'<br>Reverse 5'-TCCTGTCTAACTCATCTTTCATCG-10
ACT7	AT5G09810	Member of Actin gene family	Forward 5'-TGTTCCCAGGAATTGCTGACCGTA-3'<br>Reverse 5'-TTGATCTTCATGCTGCTAGGTGCA-3
